# Small but versatile: the extraordinary functional and structural diversity of the β-grasp fold

**DOI:** 10.1186/1745-6150-2-18

**Published:** 2007-07-02

**Authors:** A Maxwell Burroughs, S Balaji, Lakshminarayan M Iyer, L Aravind

**Affiliations:** 1 National Center for Biotechnology Information, National Library of Medicine, National Institutes of Health, Bethesda, MD 20894, USA; 2 Bioinformatics Program, Boston University, Boston, MA 02215, USA

## Abstract

**Background:**

The β-grasp fold (β-GF), prototyped by ubiquitin (UB), has been recruited for a strikingly diverse range of biochemical functions. These functions include providing a scaffold for different enzymatic active sites (e.g. NUDIX phosphohydrolases) and iron-sulfur clusters, RNA-soluble-ligand and co-factor-binding, sulfur transfer, adaptor functions in signaling, assembly of macromolecular complexes and post-translational protein modification. To understand the basis for the functional versatility of this small fold we undertook a comprehensive sequence-structure analysis of the fold and developed a natural classification for its members.

**Results:**

As a result we were able to define the core distinguishing features of the fold and numerous elaborations, including several previously unrecognized variants. Systematic analysis of all known interactions of the fold showed that its manifold functional abilities arise primarily from the prominent β-sheet, which provides an exposed surface for diverse interactions or additionally, by forming open barrel-like structures. We show that in the β-GF both enzymatic activities and the binding of diverse co-factors (e.g. molybdopterin) have independently evolved on at least three occasions each, and iron-sulfur-cluster-binding on at least two independent occasions. Our analysis identified multiple previously unknown large monophyletic assemblages within the β-GF, including one which unifies versions found in the fasciclin-1 superfamily, the ribosomal protein L25, the phosphoribosyl AMP cyclohydrolase (HisI) and glutamine synthetase. We also uncovered several new groups of β-GF domains including a domain found in bacterial flagellar and fimbrial assembly components, and 5 new UB-like domains in the eukaryotes.

**Conclusion:**

Evolutionary reconstruction indicates that the β-GF had differentiated into at least 7 distinct lineages by the time of the last universal common ancestor of all extant organisms, encompassing much of the structural diversity observed in extant versions of the fold. The earliest β-GF members were probably involved in RNA metabolism and subsequently radiated into various functional niches. Most of the structural diversification occurred in the prokaryotes, whereas the eukaryotic phase was mainly marked by a specific expansion of the ubiquitin-like β-GF members. The eukaryotic UB superfamily diversified into at least 67 distinct families, of which at least 19–20 families were already present in the eukaryotic common ancestor, including several protein and one lipid conjugated forms. Another key aspect of the eukaryotic phase of evolution of the β-GF was the dramatic increase in domain architectural complexity of proteins related to the expansion of UB-like domains in numerous adaptor roles.

**Reviewers:**

This article was reviewed by Igor Zhulin, Arcady Mushegian and Frank Eisenhaber.

## Background

**Figure 1:**
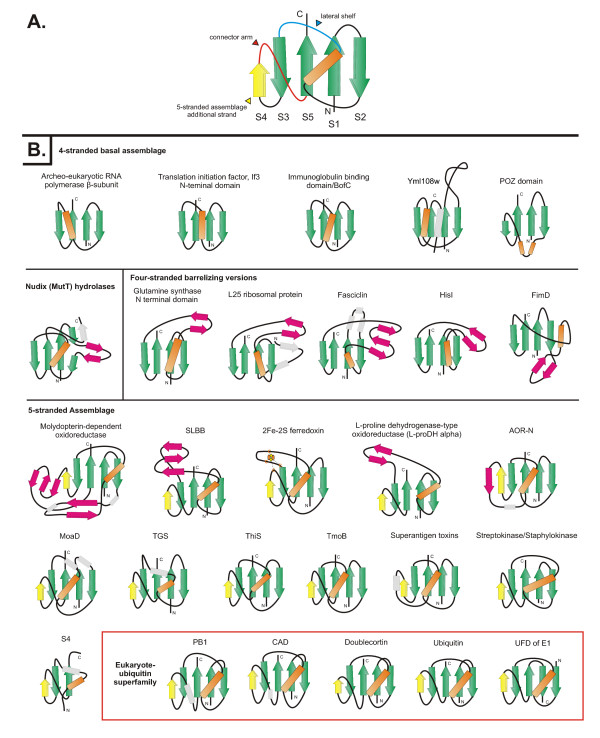
Topology diagrams of selected β-GF members. A generalized representative is shown in (A) with the key structural features found in certain lineages of the fold labeled, while (B) depicts idealized versions of specific lineages, the names of which are given above the diagrams. Strands are shown as arrows with the arrowhead at the C-terminal end. Strands belonging to the 4-stranded β-GF core are colored green, the additional strand found in the 5-stranded assemblage is colored yellow, strands forming a conserved insert within the β-GF scaffold are colored magenta, and other strands specific to a certain lineage are colored grey and outlined with a broken line. Helices are depicted as rectangles, with the core absolutely conserved helix colored orange and other helices specific to a certain lineage colored grey and outlined with a broken line. The diagrams are grouped and labeled in a manner consistent with the structural classes described in the text, with members of the eukaryotic UB-like superfamily nested within other members of the 5-stranded assemblage. The 2Fe-2S cluster of the ferredoxins is shown as four small ovals bound to cysteine residues represented by the letter "C".

The discovery of covalent modification of eukaryotic proteins by the conjugation of ubiquitin to the ε-amino groups of target lysines has spawned some of the most exciting directions of research in current molecular biology [1-3]. Ubiquitin (Ub) itself is a small polypeptide of 76 residues, and its crystal structure revealed a distinctive fold dominated by a β-sheet with 5 anti-parallel β-strands and a single helical segment [4,5] (Figure 1A). Pioneering investigations of Kraulis, Overington and Murzin showed that this fold was not unique to Ub, but was also present in several other proteins with biologically distinct functions. These included the staphylococcal enterotoxin B, the streptococcal immunoglobulin (Ig)-binding protein G and 2Fe-2S ferredoxins [6-8]. The common fold shared by these proteins was termed the β-grasp, because the β-sheet appears to grasp the helical segment in this domain [7]. These early studies provided the first indications that, despite its small size, the β-grasp fold (β-GF) might serve as a multi-functional scaffold in diverse biological contexts.

The centrality of Ub conjugation in eukaryotic molecular biology has led to numerous investigations on Ub and Ub-related domains [9,10]. These studies have resulted in a large body of data on the properties of the Ub-like versions of the β-GF. The key emerging findings were that several other Ub-like proteins (Ubl), such as Urm1 [11], Apg12 [12], Nedd8 [13], and SUMO [14,15] are also covalently linked to target polypeptides, just as Ub itself. In contrast, some Ub-related domains, like the Ubx domain or Ub-like domains of IκB kinases, play adaptor roles in Ub-signaling [16-19]. These studies also showed that eukaryotes possess a distinctive enzymatic apparatus for Ub-modification, comprised of a cascade of three enzymes: E1, E2 and E3. These enzymes successively activated Ub/Ubls for transfer using the free energy derived from ATP hydrolysis, relayed it via thiocarboxylate linkages involving the C-terminal residue of Ub/Ubls, and finally transferred it to lysines on target polypeptides [1,10,20-22]. Eukaryotes were also shown to contain an elaborate apparatus for removal of covalently linked Ub/Ubls and proteasomal degradation of Ub-modified proteins [23-27].

Concomitantly, structural studies also uncovered several new versions of the β-GF in a variety of domains, greatly widening its horizon of biological functions. Examples of such β-GF domains are: 1) the TGS domain, an RNA-binding domain found in aminoacyl tRNA synthetases and other translation regulators (PDB: 1QF6 [28,29]). 2) The doublecortin (DCX) (PDB: 1MJD [30]), RA (PDB: 1C1Y [31]), PB1 (PDB: 1IPG [32]), and FERM N-terminal domains (PDB: 1EF1 [33]), which function as adaptors in animal signaling proteins and apoptosis regulators by mediating protein-protein interactions. 3) The soluble ligand-binding β-GF (SLBB) domain involved in binding vitamin B_12 _and other solutes in animals and bacteria (PDB: 2BBC, 2FUGS [34-36]). 4) Various toxins related to the staphylococcal enterotoxin B including superantigens involved in the toxic shock syndrome (PDB: 1ESF [37]). 5) Functionally obscure subunits of various enzymatic complexes, like TmoB of the aromatic monooxygenase oxygenase complex (PDB: 1T0S [38]) and RnfH of the Rnf dehydrogenases [39]. 6) Conserved domains, perhaps involved in RNA binding, in the archaeo-eukaryotic RNA polymerase RPB2 subunit [40] and bacterial translation initiation factor IF3 (PDB: 1TIF [41-43]). 7) Staphylokinases and streptokinases which are fibrinolytic enzymes of low GC Gram-positive bacteria (PDB: 2SAK [44]). 8) MutT/nudix enzymes – a group of phosphohydrolases acting on diverse substrates [45]. These observations suggested that the β-GF is indeed a widely utilized structural scaffold, with an underappreciated versatility and an evolutionary history rich in adaptive radiations.

One notable evolutionary question in this regard was the origin of eukaryotic Ub and its relationships to other domains with the β-GF. The first major advances in this direction came with the identification of the sulfur transfer proteins, ThiS and MoaD, respectively involved in thiamine and Molybdenum cofactor (MoCo) biosynthesis, which contained β-GFs closely related to Ub [46,47]. Furthermore, it was demonstrated that their C-terminal residues formed thiocarboxylates, just like Ub, and this was catalyzed by enzymes (ThiF and MoeB), which are very similar to the E1 enzymes involved in Ub-conjugation [46-50]. More recently, research from our group showed that the Ub-conjugation systems might not be an exclusive feature of eukaryotes. Proteins with Ub-like β-GF domains, and functionally linked enzymes related to E1, E2 and deubiquitinating peptidases of the JAB domain superfamily were found in several, phylogenetically diverse bacteria. We presented evidence that though some of these systems are likely to be involved in sulfur transfer reactions in metabolite biosynthesis, akin to ThiS and MoaD, others might potentially function as *bona fide *conjugation systems that transfer β-GF proteins to target polypeptides [39]. Hence, the eukaryotic Ub-conjugation system might have evolved from more ancient precursors that were present in bacteria prior to the origin of eukaryotes.

With some clarity emerging on issue of the origin of Ub/Ubls and the associated biochemical networks, we sought to investigate the broader issue of the adaptive radiations of the entire β-GF. In particular we were interested in a number of problems from structural and evolutionary stand points: 1) Establishing the entire gamut of structural and topological variations that have emerged in the β-GF. 2) Identifying any unifying structural themes that might exist across most or all functionally diverse versions of the fold. 3) Determination of the lineage-specific sequence-structure correlates for the varied functional adaptations of the β-GF. 4) Developing a higher order evolutionary classification for the β-GF and using it as a scaffold to identify the major temporal phases of adaptive radiation. 5) Identifying instances of drastic shifts in biological or biochemical functions in specific monophyletic lineages of the β-GF. One example of such a functional shift is seen in the evolution of the classical Ub-like proteins, where a unique post-translational modification system emerged from a core metabolic sulfur transfer system. 6) Identifying previously unrecognized members of the fold, if any, and thereby expanding the functional spectrum or providing a rationale for function prediction of uncharacterized members of the fold. 7) We also hoped that the β-GF might provide a model for understanding the more general problem of how certain small protein folds tend to be extensively deployed in a whole diversity of functional contexts.

In this article we present the results of our systematic analysis of the β-GF with the objective of addressing the above points.

## Results and Discussion

### Identification of β-GF domains

As the β-GF is small in size and its representatives very divergent, it is not possible to exhaustively identify all members through sequence or structure similarity searches initiated from a single starting point. Accordingly, we used a multi-pronged strategy of sequence, structure, and topological similarity searches. As a starting point, we used all the currently available structures of β-GF proteins from the Protein Data Bank (PDB) [51]. This set was compiled by collecting all structures already classified under the β-GF in the SCOP database [6], their relatives from the PDB database that are not present in SCOP, and new versions which were detected in our recent studies [34,39]. These representatives were used as seeds for initiating sequence profile searches of the NCBI NR (non-redundant) database with the PSI-BLAST program [52] (see materials and methods for details). Statistically significant hits (e < 0.01) recovered in these searches were used to generate alignments for further HMM searches of individual genome databases and representatives used for transitive PSI-BLAST searches of the NR database. All newly-identified clusters of domains distinct from previously identified sequence families containing the β-GF were aligned and used to predict secondary structure with the JPRED program [53]. The predicted secondary structure and the conservation pattern were superimposed onto the secondary structure and conservation patterns of the known β-GF sequence families to ascertain the validity of the newly-detected versions (see Additional file 1 for alignments and complete list of recovered sequences).

All available structures of *bona fide *β-GF domains were compared in order to establish a unique core template topology that discriminated the β-GF from all other folds (Figure 1A; Table 1; see below for further details). Then the representative structures of β-GF domains were used as queries to search a local current version of the PDB database for structurally similar domains using the DALILITE program [54,55]. All hits were evaluated through reciprocal DALILITE searches of the PDB database to determine if their best matches included any known β-GF proteins. The hits were also further evaluated for congruence to the unique topological template. In addition to the match to the core structural template, we also systematically documented all unique features of each newly-detected structure. Through these searches we were able to identify around ten previously unknown families/superfamilies of domains containing the β-GF, including certain structurally distinctive variants. Comparisons of the distributions of previously characterized globular domains in proteins from sequenced genomes suggests that our procedures have identified a major fraction of conserved lineages of the β-GF.

**Table 1:** Secondary structure features of major β-GF structural categories.

Higher-order Classification	Lineage Name	Secondary Structural Features Common to the β-GF Fold^1^												
		
		S1	L1	S2	L2	H	L3/LS	S3	L4	S4	L5/CA	S5	tail	notes
Basal 4-stranded versions of the β-GF	IF3-N	S1	--	S2	--	H	--	S3	--	O	O	S5	--	
	Archeo-eukaryotic RNA poly. β-subunit	S1	--	S2	--	H	--	S3	--	O	O	S5	--	
Sporadically-distributed 4-stranded versions	Yml108w	S1	cc	S2	--	H	--	S3	--	O	O	S5	h	
	BofC	S1	--	S2	--	H	--	S3	--	O	O	S5	--	
	Immunoglobulin-binding	S1	--	S2	--	H	--	S3	--	O	O	S5	--	
	POZ	S1	--	S2	--	H	h	S3	--	O	O	S5	--	
Nudix superfamily	Nudix (MutT)	S1	--	S(ee)2	--	H	*	S3	--	O	O	S5	e	
Fasciclin-like assemblage	L25	S1	--	S2	--	H	ee*	S3	--	O	O	S5	--	3
	glutamine synthetase N-terminal	S1	--	S2	--	H	eee*	S3	--	O	O	S5	--	3
	fasciclin	S1	hhh	S2	--	H	ee*	S3	--	O	O	S5	--	3
	phosphoribosyl AMP cyclohydrolase (HisI)	S1	--	S2	--	H	ee*	S3	--	O	O	S5	--	3,4
5-stranded assemblage: classical 5-stranded clade	MoaD	S1	H	S2	--	H	h**	S3	--	S4	*	S5	--	
	ThiS	S1	--	S2	--	H	*	S3	--	S4	*	S5	--	
	TmoB	S1	--	S2	--	H	*	S3	--	S4	*	S5	--	
	Superantigen	S1	--	S2	--	H	*	S3	--	S4	h*	S5	--	
	Strepto/Staphylokinase	S1	--	S2	--	H	*	S3	--	S4	*	S5	--	
	YukD	S1	--	S2	--	H	*	S3	--	S4	*	S5	--	
	TGS	S1	--	S2	--	H	h*	S3	--	S4	*	S5	--	
	Aldehyde OR^2 ^N-terminal domain	S1	--	S2	--	H	*	S3	--	S4	eh*	S5	--	
5-stranded assemblage: Selected eukaryote UB-like superfamily members	classic UB-like	S1	--	S2	--	H	*	S3	--	S4	*	S5	--	
	PB1	S1	--	S2	--	H	*	S3	--	S4	h*	S5	--	
	CAD/Doublecortin (DCX)	S1	--	S2	--	H	*	S3	--	S4	[h]*	S5	--	6
	RA	S1	--	S2	--	H	*	S3	--	S4	h*	S5	--	
	Elongin	S1	--	S2	--	H	*	S3	--	S4	*	S5	--	
	UBX	S1	--	S2	--	H	*	S3	--	S4	*	S5	--	
	E1/UFD	O	--	S2	--	H	*	S3	--	S4	*	S5	S6	7
5-stranded assemblage: soluble ligand binding or metal ion chelating clade	molydopterin-dependent oxidoreductase	S1	--	S2	hehee	H	*	S3	--	S4	eee*	S5	--	
	SLBB: Nqo1-type	S1	--	S2	--	H	*	S3	--	S4	hh*	S5	--	5
	SLBB: transcobalamin-type	S1	--	S2	--	H	eee*	S3	--	S4	*	S5	--	
	2Fe-2S ferredoxin	S1	--	S2	--	H	cc*	S3	--	S4	*	S5	--	
	L-proline DH-like OR^2 ^N-terminal domain	S1	--	S2	--	H	ee*	S3	--	S4	*	S5	--	
Miscellaneous	WWE	S1	--	S2	--	H	e*	S3	--	O	O	S5	e	8
	FimD N-terminal	S1	--	S2	ee	H	*	S3	--	O	O	S5	--	
	S4	O	O	O	O	H	h*	S3	--	S4	*	S5	--	

**Figure 2:**
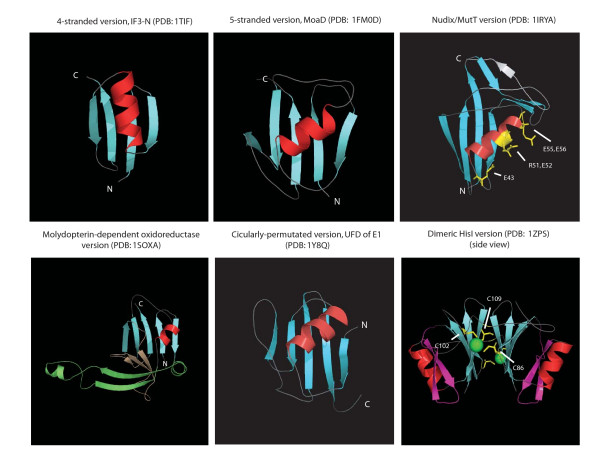
Cartoon representations of distinct β-GF domains. Critical residues in MutT and HisI that are involved in enzyme catalysis are also shown.

1. S: Strand, L: Loop, H: Helix, LS: Lateral Shelf, CA: Connector Arm, O: absence of given feature, --: presence of a loop feature, *: presence of LS or CA, h: insert in helical conformation, e: insert in extended conformation (strand-like), cc: long coil insert.

2. OR: oxidoreductase.

3. Versions form barrel through insertion of strands at the lateral shelf.

4. Barrel is less pronounced in this version, strands are inserted more upstream relative to the other 3 versions.

5. Two small helices are present in ascending arm.

6. Single helix found at ascending arm in several members.

7. Circular permutation results in new connections between strands; the S1 strand is found at C-terminus (See Figures 1, 2).

8. Additional strand at tail inserted between S1 and S5; lateral shelf forms strand that also stacks with central sheet.

### Core conserved topology, structural variation, and derivatives of the β-GF

A comparison of the available β-GF structures revealed a common core of 4 strands forming an anti-parallel sheet, and a single helical region (see Table 1, Fig. 1A). The characteristic topological feature is that the first and last strands are adjacent and parallel to each other, and the remaining two strands of the conserved core are anti-parallel and flank the former two strands on either side. The first and last strands are invariably located in the center of the sheet with a cross-over occurring via the single helical element. This helical region is packed against one face of the sheet, typically leaving the other face exposed. The chief interacting positions between sheet and the helical segment and the pattern of key stabilizing hydrophobic interactions are conserved throughout the fold, supporting its monophyletic origin. The β-GF domains found in IF3 and the second largest subunit (β-subunit orthologs) of the archaeo-eukaryotic RNA polymerase more or less correspond to this conserved core (Figure 1B). Several β-GF domains display simple structural elaborations of this basic 4-stranded core. The simplest of these is the seen in a small family of yeast proteins typified by Yml108w from *S. cerevisiae *(PDB: 1N6Z [56]). This version has a large insert between the first two strands and an additional helical extension at the C-terminus (Figure 1B). Another notable variant of the basic 4-stranded form of the β-GF domain is seen in the catalytic domain of the NUDIX (MutT) hydrolases. Here, the middle of the second strand of the conserved core is interrupted by a peculiar insert that projects out to form a distinctive "outflow". This outflow often assumes a hairpin-like configuration stabilized by hydrogen bonding between segments in an extended conformation (Figure 1B).

All other versions of the β-GF are characterized by major modifications to the 4-stranded core in the form of distinct inserts that add new secondary structure elements. The first of these is a previously uncharacterized variation containing an insertion of one or more strands between the helical segment and strand 3. The conserved inserted strand seen in all domains with this version forms a hairpin with the connector segment between the helical segment and strand 3 which also assumes an extended conformation. This hairpin, together with any additional strands in the insert results in these versions of the fold assuming barrel-like structures with differing degrees of openness (Figure 1, Table 1). Examples of this version of the β-GF domain are observed in the ribosomal protein L25 (PDB: 1B75 [57]), fasciclin (PDB: 1O70 [58]), and glutamine synthetase (PDB: 1LGR, 2GLS [59,60]). We uncovered yet another novel variant of the β-GF in the N-terminal domain of the periplasmic pilus assembly protein FimD (PDB: 1ZE3, chain D [61]). This version is typified by a unique insert N-terminal to the helical segment which results in the formation of a barrel-like configuration comparable to the above structural variants.

The most common version of the β-GF is typified by the presence of an additional strand that packs against the conserved third strand at the margin of the core β-sheet. The acquisition of this additional strand has resulted in the emergence of a connector arm that joins it to the terminal conserved strand of the core sheet (Figure 1, Table 1). All ubiquitin-like β-GF domains, including sulfur carrier proteins like MoaD and ThiS, contain this 5-stranded version of the fold. The connector arm is variable in structure and length and assumes a wide range of conformations ranging from coils to structured elements in different versions of the fold (Figure 1B, Table 1). A derivative of this Ub-like 5-stranded version is found as a C-terminal domain (UFD) in most eukaryotic E1 Ub-conjugating enzymes [62,63] – here a circular permutation appears to have displaced the N-terminus to the C-terminus. Given that the N- and C-terminal strands of the β-GF are adjacent to each other, the C-terminal strand in the permuted version occupies the same position as the N-terminal strand of the classical versions, but is oriented in the opposite direction (Figure 1, Table 1).

The 5-stranded versions may show further variations due to inserts at different points in the conserved core. One prominent example is the 2Fe-2S ferredoxin, which contains an insert before the third conserved strand with conserved cysteines for chelating the Fe ion. Similarly, a long insert adopting an extended conformation is observed at a comparable position in several versions of the SLBB domain [34] and the molybdopterin-dependent oxidoreductases (Figure 1B, Table 1). In the SLBB domain, the curved β-strands from the insert along with the strands of the β-GF domain core contribute to the formation of a barrel-like structure (PDB: 2BBC [34]). In the middle domain of molybdopterin-dependent oxidoreductases (PDB: 1SOX [64], chain A) there is an additional insert of 2 β-strands associated with the connector arm, which results in an even more complex 3-layered structure, with the two inserts forming a barrel-like element within it. Another previously unknown variant is seen in the N-terminal domain of the aldehyde oxidoreductases (AOR-N) (PDB: 1AOR [65]), wherein the connector arm assumes an extended conformation and packs as an additional strand at the fringe of the β-sheet adjacent to the strand-4 which is a specific feature of the 5-stranded versions (Figure 1B). In the AOR-Ns, two of these variant β-GF domains stack via the exposed surface of the β-sheet and form a 4-layered sandwich module.

Our structure similarity searches identified a few structures which, despite lacking the core conserved topology of the classical β-GF, aligned well with a part thereof. Reciprocal searches indicated that β-GF domains were the best hits for these structures. Additionally, these structures were not representatives of any other previously identified folds. These structures include the S4 RNA-binding domain (PDB: 1c05 [66]), the WWE domain (PDB: 2A90 [67]), and the POZ domain (PDB: 1BUO [68]). Previous structural studies had noted a region of local structural similarity, termed the α-L motif, between the S4 and the TGS domain [69]. Given the functional similarity (RNA-binding) and close structural congruence between the shared elements of these two domains, it is quite likely S4 domain is a degenerate variant of the 5-stranded TGS-like β-GF domain, which has emerged through partial loss of the N-terminal part of the domain including the first two strands. The WWE domain and the POZ domain are found only in eukaryotes [70], suggesting that they could have potentially emerged from pre-existing folds through rapid divergence. Given its general structural similarity with the β-GF domains, it is likely to have been derived from the 5-stranded version of this fold. The WWE domain appears to have acquired an additional strand after the terminal strand which is inserted in the middle of the core sheet. The pre-strand 3 region in this domain also adopts a peculiar structure which makes it appear very different from the classical β-GF domains. In contrast, the POZ domain appears to have been derived from a 4-stranded β-GF domain through different degrees of degradation of the penultimate strand on the fringe of the sheet.

### Natural classification of β-GF domains

**Figure 3:**
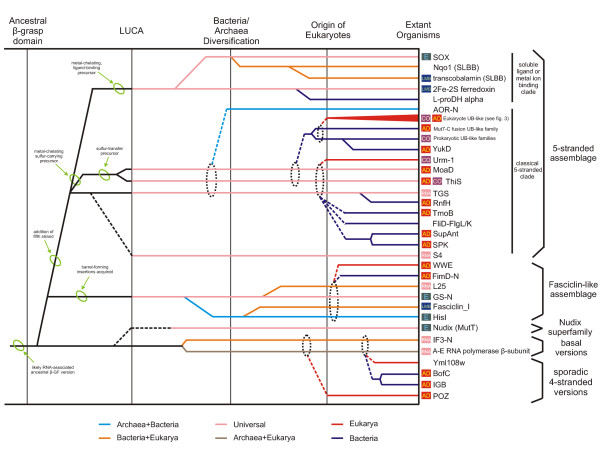
Reconstructed evolutionary history of β-grasp fold. Individual lineages are listed to the left of the figure grouped according to classifications given in the text, with their inferred evolutionary depth traced by solid horizontal lines across the relative temporal epochs representing major evolutionary transitional periods shown as vertical lines. The horizontal lines are color-coded according to their observed phyletic distributions, the key for this coloring scheme is given at the bottom of the figure. Dashed lines indicate uncertainty in terms of the origins of a lineage, while grey ellipses group lineages of relatively restricted phyletic distribution with more broadly distributed lineages, indicating that the former likely underwent rapid divergence from the latter. Major predicted structural/functional transitions of the fold are marked by green ellipses with a brief description given. Colored, labeled squares immediately to the left of the lineage names represent broad functional categories: E, enzymatic activity; LMB, ligand or metal-binding; CO, conjugated versions; AD, mediator of protein-protein interactions; RNA, RNA metabolism-related.

In order to address the prime evolutionary questions about the β-GF, we attempted to construct a classification that most closely approximates the higher-order evolutionary relationships of the members of this fold. The small size of the majority of the versions of this domain often precludes sufficient resolution of relationships using conventional phylogenetic tree methods, sometimes even within superfamilies that display significant sequence similarity. This difficulty is further compounded by the extreme sequence divergence even between versions having highly similar tertiary structures (e.g. ubiquitin and ThiS). Hence, we had to rely to a greater extent on structure similarity-based clustering, shared derived structural characters, and phyletic patterns of sequence superfamilies to reconstruct the evolutionary history. Thus, we produced the classification using the following general steps: 1) sequence similarity-based clustering with the BLASTCLUST program [71] helped in identifying the cores of all major sequence families of β-GF domains. 2) Subsequent comparison of the individual sequence conservation profiles led to the establishment of the most inclusive higher-order assemblages of these families (termed superfamilies) based on shared derived features. 3) The next level of relationships beyond what could be resolved through sequence comparisons was established using structural similarity. This was done both by constructing distance trees based on pairwise Z-scores for structure similarity and deriving the most parsimonious tree based on shared structural features (see Table 1 for major structural features). This procedure, while allowing reasonable resolution of the higher-order relationships, might on occasions produce relatively flat hierarchies for lower-level clusters where none of the methods offer reliable resolution of relationships. A summary of this classification is presented in Figures 2, 3 and Additional file 2.

#### Basal versions and other sporadically distributed 4-stranded versions of the β-GF

The above analysis of the structural diversity of the fold suggests that the 4-stranded version is the simplest form from which all other versions could have been derived through accretion of inserts and additional secondary structure elements. Two structurally close superfamilies of the 4-stranded β-GF domain, namely the IF3-N and the archaeo-eukaryotic RNA polymerase domain, are respectively universally conserved in the bacterial and archaeal-eukaryotic branches of life. This, taken together with their shared general functional connection to RNA metabolism, suggests that they arose from a similarly structured precursor that can be traced back to the last universal common ancestor (LUCA). This structurally simple representative of the β-GF is likely to represent one of the most basal lineages of the fold. The remaining sequence clusters BofC, yeast Yml108w, and immunoglobulin-binding proteins of low GC Gram-positive bacteria with structurally comparable, simple 4-stranded β-GF domains show extremely limited phyletic patterns (Additional file 2), suggesting a probable recent derivation from the more ancient versions. The versions in the Ig-binding proteins and BofC are restricted to Gram-positive bacteria, and the former might have been derived in pathogenic forms from BofC, which is a secreted developmental signaling molecule widely distributed in free-living Gram-positive bacteria [72,73]. The eukaryote-specific POZ domain might represent another derivative of a more widely-distributed 4-stranded version, which has accreted an additional C-terminal helical bundle to form a distinctive globular structure (Figures 1, 2 and Table 1).

#### The Nudix (MutT) superfamily

The remaining versions of the β-GF fold appear to form a monophyletic clade unified by the presence of an ancestral "lateral shelf" or "flange" that forms an extended connector between the helical segment and the remaining portion of the sheet after the topological cross-over (Figure 3 and Table 1). Of these versions, the Nudix superfamily appears to be one of the early branches given that its β-sheet retains the ancestral 4-stranded core. All members of this superfamily share the above-described insert or "outflow" in the middle of strand 2 which forms a distinctive shelf for accommodating substrates. This superfamily is also unified by the presence of a conserved PXG motif in strand-2, immediately after the "outflow", and a unique constellation of conserved residues in the helical segment which form the phosphohydrolase active site [74,75]. The Nudix superfamily represents a rare instance of adaptation of the β-GF as a scaffold for catalytic activity. Its phyletic patterns suggest an ancestral presence in all three superkingdoms implying that it might have been present in the LUCA (Figure 3, Additional file 2).

#### Fasciclin-like assemblage

A structurally distinct subgroup of β-GF domains which was uncovered as a result of our analysis unifies previously unrecognized versions of the fold, namely the fasciclin domain (PDB: 1O70 [58]), the ribosomal protein L25 (PDB: 1B75 [57]), and the phosphoribosyl AMP cyclohydrolase (HisI) (PDB: 1ZPS [76]) with the glutamine synthetase N-terminal domain. The unique insert and associated structural peculiarities such as the barrel-like configuration shared by these domains strongly suggests that they form a higher-order monophyletic cluster within the β-GF termed the fasciclin-like assemblage. The similar ligand-interaction patterns seen in most of these lineages also support the monophyly of this assemblage (see below for details). Most characterized sequence superfamilies within this assemblage appear to bind small molecules or soluble ligands. The fasciclin domain binds sugar moieties of cell-surface glycoproteins [58], the HisI domain binds phosphoribosyl AMP [77], and the glutamine synthetase N-terminal domain contributes to the substrate binding pocket of the enzyme [59]. The L25 domain binds 5S RNA (PDB: 1DFU [78]), although there is no evidence that it does so in a comparable manner as the other members of this assemblage. Given the above observations, it is possible that the ancestral version of this assemblage had small-molecule binding capabilities. Despite the distinctive structural innovations, the conserved core of the β-GF domain in this assemblage is a 4-stranded version with a "lateral shelf" suggesting that it represents an early branch of the clade unified by the latter derived feature (Figure 3). Of the sequence superfamilies of this assemblage, the glutamine synthetase N-terminal domain is traceable to LUCA. Hence, the fasciclin-like version of the β-GF domain might have diverged from other major lineages of the fold prior to LUCA.

The FimD superfamily, while containing a unique structural variant of the fold, shows greatest structural similarity to the above assemblage. Its phyletic pattern is limited, being found only in proteobacteria and deinococci (Additional file 2). Thus, it could have been derived from the above assemblage in a lineage-specific manner.

#### The 5-stranded assemblage

The 5-stranded assemblage is unified by the addition of the fifth strand to the core sheet and the consequent emergence of the "connector arm" linking the additional strand to the terminal strand (Figure 1A). The strong conservation of this unique structural feature, in conjunction with the exclusive grouping of these versions in structure similarity-based clustering, suggests that they form a monophyletic assemblage. This version of the fold is most prevalent, both in terms of number of distinct superfamilies contained within it and universal representation found across all life forms. At least 4 monophyletic lineages of this assembly, namely the TGS domain, the ThiS and MoaD proteins, and the 2Fe-2S ferredoxins can be traced to LUCA. Beyond these, there are several lineages that are conserved in a single superkingdom or distributed more sporadically within a superkingdom. On the whole, two major clades can be recognized within the 5-stranded assemblage. The first of these, termed ***the classical 5-stranded clade***, unites the three ancient lineages TGS, ThiS, and MoaD and several other closely-related versions. This clade is also supported by the presence of a highly conserved alcoholic residue at the transition between the N-terminal hairpins and the helical segment of the fold [39]. The UB-like β-GF domains are derived from the ThiS and MoaD-like versions and comprise the most diverse superfamily within the classical 5-stranded clade.

##### Eukaryotic representatives of the UB-like superfamily β-GF domains

In eukaryotes, this superfamily has undergone explosive diversification with at least 19–20 distinct families which can be traced back to the last eukaryotic common ancestor (LECA). These families include six conjugated versions (ubiquitin, Urm1, Apg8/Aut7, Apg12, Ufm1 and SUMO/SMT3) [79,80] and several known or predicted to function as adapters in multi-domain proteins, like the tubulin cofactor B (TBCB) [81], Ub/Ubl conjugating E1 enzymes [62,63] and phosphatidyl-inositol 3 kinase (PI3K) [82]. Overall, in the course of eukaryotic evolution, at least 67 distinct sequence families appear to have emerged within this superfamily with some restricted to particular eukaryotic kingdoms like animals or plants. We identified several previously uncharacterized eukaryotic families such as NPL4p, the UB-like domains of the BMI1/Posterior Sex Combs family of chromatin associated E3 ligases, a family with the UB-like domain fused to a cytochrome b5 domain, and the auxin response factor (BIPOSTO) in plants (see Additional file 1 for alignments). On the whole, comparisons of sequence conservation profiles showed that β-GF domains related to the classical ubiquitin domain form a large monophyletic assemblage within the superfamily, including several distinct families such as Nedd8, SUMO, ubiquitin, NPL4, BAG, the Ubx domain, the tubulin co-factors or chaperones (TBCB and TBCE), Bat3/Dsk and Apg12/Gate16 (Fig. 3). The circularly permuted C-terminal UFD of eukaryotic E1s, which distinguishes them from the prokaryotic E1-related enzymes, also appears to have been derived from this lineage. Sequence comparisons also showed that the RA, FERM N-terminal module, and PI3K adapter domain families form another distinct higher-order monophyletic lineage. The remaining lineages typified by ECR1/UBA1 and BM-002, while structurally close to the rest, formed distinct sequence families that could not be placed into the any of the above larger assemblages of families (see Additional file 2 for details).

##### Bacterial representatives of the UB-like superfamily and the classical 5-stranded assemblage

In bacteria, Ub-like superfamily includes several sporadically distributed UB-like families which have been previously described in considerable detail [39]. Several other sporadic bacterial lineages also belong to the classical 5-stranded clade, such as the fibrinolytic adapters of several Gram-positive bacteria (e.g. streptokinase), the superantigen/toxin domains, the RnfH proteins and subunits of aromatic compound monooxygenases like TmoB. Our searches also identified a previously unknown version of the classical 5-stranded clade in a group of bacterial flagellar assembly proteins typified by FliD, FlgL and FlgK, and related bacteriophage-tail proteins found in a range of Mu-like caudoviruses (see Additional file 1). Sequence searches indicate that RnfH is closest to the TGS domains and is likely to be an offshoot of that superfamily (Fig. 3). The superantigen/toxin versions and the streptokinase/staphylokinases appear to form a monophyletic cluster, as they are both secreted versions and interact with substrates similarly (See below). However, barring RnfH, the exact relationships of these more sporadic bacterial lineages to the more ancient lineages of the classical 5-stranded clade remain unclear.

##### The soluble ligand or metal-binding clade of the 5-stranded assemblage

The second major clade of the 5-stranded assemblage unifies a group of β-GF domains whose interrelationships were previously unknown. This clade is unified by the presence of a set of inserts that are associated with binding soluble ligands or chelating metal ions. While the inserts themselves are poorly conserved in sequence, their position, especially in relation to the bound ion or ligand, is well conserved. The main sequence superfamilies in this clade are the 2Fe-2S ferredoxins, the SLBB domains, and the molybdopterin-dependent oxidoreductase domains. As recently shown, the SLBB superfamily is of bacterial provenance [34]. The molybdopterin-dependent oxidoreductases, typified by the sulfite oxidase (SOX), are widely distributed in all the three superkingdoms but show no evidence in phylogenetic analysis for being present in LUCA. Given that the eukaryotic versions localize to the mitochondrion [83], they appear to have probably been derived from the bacterial progenitor of the mitochondria. The N-terminal domain of the L-proline dehydrogenase-type oxidoreductase (PDB: 1Y56 [84]) is another family of proteins belonging to this clade of the 5-stranded assemblage. Sequence profile analysis showed a statistically significant relationship between these domains and the 2Fe-2S ferredoxins, suggesting that they belong to the same superfamily. They appear to have been derived from the more universally distributed 2Fe-2S ferredoxins through loss of the metal-chelating conserved cysteines relatively early in bacterial evolution.

##### The N-terminal module of the aldehyde oxidoreductases

A distinctive superfamily of the 5-stranded assemblage that we discovered in our analysis was the N-terminal module of the aldehyde oxidoreductase (AOR-N) (PDB: 1AOR [65]) that contains two tandem, distantly related copies of the β-fold. These are unified by the modified structure of their connector arm, ligand-binding and dimerization pattern. This structural modification makes it difficult to identify their affinities to other members of the 5-stranded assemblage. It should be noted that they lack any unique structure or sequence feature unifying them to the sulfite oxidase-like molybdopterin-binding β-GF domains. Hence, it is possible that they arose from a MoaD-like precursor that evolved an ability to bind metallopterins specifically (See below). Phyletic patterns indicate a potential bacterial origin for this superfamily. The above-mentioned structural similarity of the universally distributed S4 RNA-binding domain with the TGS domain suggests that the former might be another highly divergent lineage that was derived from a TGS-like classical 5-stranded β-GF domain prior to LUCA.

### The relative timeline of major adaptive radiations and functional transitions of the β-GF domains

#### The pre-LUCA phase and inference of the ancestral function of the β-GF

The inference of at least 7 β-GF or β-GF-derived (the S4 domain) lineages in LUCA suggests that there was a major diversification of the fold even before LUCA (Figure 3). In structural terms, the inferred representatives in LUCA span all major variants of the fold, from the simplest 4-stranded versions to the barrel-like forms (GS-N domain) to simple and elaborated versions the 5-stranded form. This suggests that the major structural variations were already in place as a result of the early diversification events of the pre-LUCA phase. In functional terms, versions close to the primitive state of both the 4- and 5-stranded forms, the RNA polymerase/IF3-N domain and the TGS domain, respectively, as well as the possible β-GF derivative, the S4 domain, have functions related to RNA metabolism or RNA-binding [29,43,85]. Even members of the Nudix clade are known to interact with nucleic acids or chemically-related molecules such as nucleoside diphosphate derivatives [74]. RNA metabolism-associated functions are also sporadically observed in later-derived lineages such as the L25 ribosomal proteins in the fasciclin-like assemblage, the family of prokaryotic UB-related domains fused to the Mut-7C-like RNAses [39], and several eukaryotic UB-like domains like those found in eIF3 p135/Clu-1 (see Additional file 1 for an alignment), RBBP6 (DWNN domain) [86], and prp21/Splicing factor 3 [87]. Given that the at least 4 of the seven main lineages traceable to LUCA, including some of the inferred basal lineages, have a RNA/ribonucleoprotein associated role, it appears likely that the ancestral version of the β-GF was probably involved in RNA-binding. The distribution of RNA-related roles (Fig. 3, Fig, 4) implies that this function seems to have been retained or re-acquired in some sense in several later derived versions of the fold.

**Figure 4:**
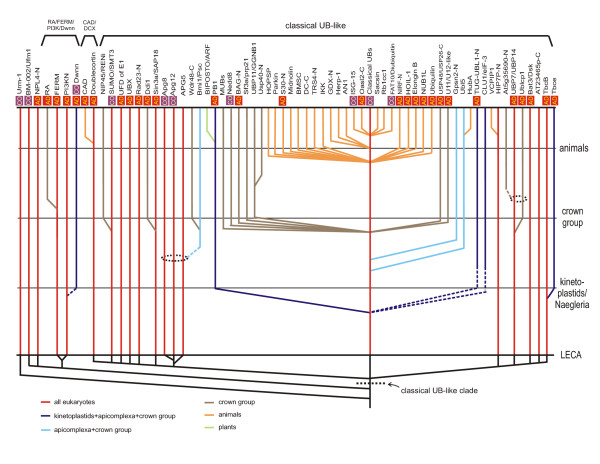
Reconstructed evolutionary history of eukaryotic ubiquitin superfamily. Similar to Figure 3, however, major evolutionary transitions are now shown as horizontal lines and the maximum depth to which these individual lineages can be traced is now shown with solid vertical lines. Functional categories are the same as described in Figure 3.

A corollary to the inference of the ancestral function of the fold is that there were major functional innovations even in the pre-LUCA period. These are most prominently seen in the 5-stranded assemblage, and appear to be associated with the emergence of distinctive roles in sulfur delivery and scaffolding of Fe-S clusters. Previous observations have shown biochemical links between the formation of metal-sulfur clusters and sulfur transfer, including pathways in which ThiS and MoaD-like proteins participate [88]. This observation raises the intriguing possibility that the earliest functional shift involved recruitment of a 5-stranded β-GF domain for a shared general role in both sulfur transfer and generation of Fe-S clusters. It is quite possible that the subsequent specialization of such a generic precursor spawned the two paralogous families of sulfur transfer proteins (MoaD and ThiS) on one hand and the 2Fe-2S ferredoxins on the other. The rise of the 2Fe-2S ferredoxins probably coincided with the emergence of the precursors of the electron transfer chains of respiratory metabolism. The early divergence of MoaD and ThiS suggests that some basic aspects of the biosynthetic pathways for complex sulfur-containing metabolites like molybdenum/tungsten cofactor and thiamine evolved prior to LUCA.

#### The post-LUCA phase: the prokaryotic superkingdoms

The emergence of the two prokaryotic superkingdoms, the archaea and bacteria, was marked by numerous superkingdom-specific innovations. Several of these innovations appear to have happened early in the history of the bacteria followed by multiple lateral transfers to the archaea. Likewise, innovations occurring in bacteria were also transferred to eukaryotes both during the primary endosymbiotic event and sporadically through later transfers. Members performing some form of most of the biochemical functions observed in extant representatives of the fold emerged in course of the post-LUCA diversification in bacteria. In certain cases there were no major shifts in basic biochemical activity but only an expansion of the range of specific biological contexts in which these activities were deployed. These included new RNA-binding/ribonucleoprotein-related contexts emerging within diverse branches of the clade (e.g. L25 in the fasciclin-like assemblage and the prokaryotic UB-like family fused to Mut-7C RNAse) or adaptation of ThiS/MoaD-type proteins in sulfur transfer systems related to synthesis of lineage-specific metabolites [89]. The principal, early functional innovations in the prokaryotic radiations were the independent acquisition of multiple small molecule/solute-binding capabilities across distant members of the fold, as seen in the SLBB, fasciclin, and AOR-N domains. Another notable feature of this evolutionary phase was the emergence of at least three catalytic versions amongst phylogenetically distant assemblages of the fold. The phosphoribosyl AMP cyclohydrolase of the fascilin-like assemblage and molybdopterin-dependent oxidoreductase domain related to the 2Fe-2S ferredoxins and SLBB domains are sister groups of the small-molecule binding versions. This suggests that the transition to catalysis probably occurred from an ancestral soluble ligand-binding state. However, emergence of catalysis in the Nudix superfamily appears to be a likely extension of the original nucleic acid-binding properties of the fold.

This phase also saw the recruitment of several forms of the β-GF domain for mediating specific protein-protein interactions in the assembly or stabilization of multi-protein complexes. Different distantly related β-GF domains were recruited in the biogenetic systems of flagella and analogous structures, the pili. The FimD protein has an N-terminal β-GF domain fused to a C-terminal outer membrane-spanning domain [90]. This β-GF domain serves as an adapter to recruit the fimbrial subunit chaperone FimC while the C-terminal domain serves as a platform on which the fimbrial subunits assemble to form the pilus [91,92]. Likewise, novel versions of the classical 5-stranded β-GF domain, which we discovered in FliD and FlgL/FlgK, are likely to play roles in the assembly of flagellum (FliD) and its hook (FlgL/K), while their relatives in Mu-like bacteriophages might similarly help in assembly of the viral tail (see Additional file 1). Pathogenic bacteria appear to have sporadically adapted both 4- and 5-stranded versions in roles related to interaction with host proteins as a part of their virulence. The strepto/staphylokinases which interact with plasmin, and the superantigens which interact with vertebrate T-cell receptors [93] from the 5-stranded assemblage and the immunoglobulin-binding domains [94] of the 4-stranded assemblage appear to represent multiple convergent recruitments for virulence-related interactions. The classical 5-stranded clade in particular appears to have given rise to several lineages that seem to function as protein interaction adapters, assembly or stability factors in very different biochemical contexts. For example, the TmoB family might function in stabilizing the proteobacterial aromatic monooxygenase complex [38], different members of the RnfH family might play roles in protein stability or assembly of the Rnf oxidoreductase complex, and YukD in the assembly of the ESAT-type export systems of Firmicutes [39].

However, the most important innovation in the bacteria was the emergence of potential conjugation systems that covalently linked ubiquitin-like β-GF domains to other proteins (predecessors of the eukaryotic conjugation systems). In functional terms, this process represents a collusion of the sulfur-transfer aspect with the protein interaction function which was also widely emerging in members of the fold. The preliminary analysis of these bacterial UB-like systems suggests that they might have already acquired roles related to protein stability and signaling. The details of the bacterial antecedents of the eukaryotic UB-conjugation system have already been discussed in a recent work [39] and are not dwelt upon here.

#### The eukaryotic phase: expansion of the ubiquitin-like domains

Genomic and cell biological evidence suggests that the eukaryotes emerged as a result of a basic endosymbiotic event between a proteobacterium and an archaeon (most likely a euryarchaeon) [95-97]. Consequently, eukaryotes inherited several versions of the β-GF domain found in both their archaeal and bacterial (mitochondrial) precursors (see Figure 2 and Additional file 2). The currently available data implies that in eukaryotes there was no diversification of the β-GF domain comparable to what happened in bacterial evolution that resulted in emergence of fundamentally new biochemical activities. Eukaryotes, however, showed an explosive development of the ubiquitin-like lineage resulting in forms that occupied biological functional niches across the entire cell. Most of these functions depend on the ancient property of the classical ubiquitin-like 5-stranded version to mediate protein-protein interactions, particularly in relation to the assembly or stabilization of complexes. These functions were performed either via conjugation of UB/UBLs to target proteins and phosphatidylethanolamine, or as domains within multi-domain proteins. The biochemical diversification of the UB-like clade to perform multiple biological roles appears to have been notable even in LECA (Figure 4). These adaptations include: 1) conjugation to proteins destined for degradation (classical UB). 2) Tagging of proteins for altering interactions and localization (e.g. SUMO/SMT3) [14,15] 3) conjugation to both a protein target (Apg5p) and the amino group of the lipid phosphatidylethanolamine (Agp8p/Aut7p) in regulation of the distinctly eukaryotic process of autophagy. 4) Possible recognition of proteins with conjugated UB moieties (e.g. NPL4) [98]. 5) Binding of E2s to present them to the active site of E1s for conjugation of UB/UBls (the UFD of E1s [62,63]). 6) Assembly of tubulin polymers (TBCB) [81] and microtubule-binding (DCX domains [30]). 7) Protein-protein interactions in Ub-modification (e.g. Ub-like domains in Ub-deconjugating enzymes like Ubp7/Ubp14 and the Bmi1/Posterior Sex Combs-like E3s) and other signaling pathways (e.g. PI3 Kinase N-terminal domain) [82]. The ancestral eukaryotic member of the UB-like clade is likely to have been a conjugated version because: 1) conjugated forms are seen across the entire diversity of the eukaryotic UB-like clade, which includes at least 5 versions traceable to LECA and 2) they preserve the basic thiocarboxylate-forming chemistry seen in their even more ancient precursors like ThiS or MoaD. Given the inferred presence of multiple non-conjugated forms in LECA, multiple early functional shifts resulting in non-conjugated appear to have occurred prior to the divergence of extant eukaryotes from LECA, but after the emergence of the first eukaryotic cell.

Subsequently in eukaryotic evolution, there appear to have been several innovations of non-conjugated versions. Many of these continued to function in contexts related to UB signaling, presumably by recognizing conjugated UB moieties (Figure 4, Additional file 2). However, a few seem to have acquired entirely unrelated functions; for example, the RA domain in RAS signaling [31] and the CAD domain in apoptotic signaling [99-102]. In temporal terms, a major pre-LECA expansion resulted in at least 19–20 distinct families in the ancestor of extant eukaryotes, followed by new families like the PB1 domain sporadically appearing throughout subsequent eukaryotic evolution. A notable phase of new innovation through sequence diversification resulted in several new families (e.g. Nedd8) prior to the radiation of the eukaryotic crown group comprised of plants, slime molds, fungi, and animals. Interestingly, in the animal lineage alone, there appears to have been another massive round of diversification resulting in more than 10 distinct sequence families. The plants show a lineage expansion of a group of UB-like domains in the BIPOSTO/ARF transcriptional regulators (see Additional file 1) which emerged from the more ancient PB1 family. Thus, in general, there appears to be a correlation between the emergence of new UB-like families and that of multi-domain proteins in the signaling systems of crown group eukaryotes, especially animals [103]. Parallel to this expansion of UB-like domains in eukaryotes, there was also an expansion of other components of the UB-conjugation system such as E1, E2, and E3 enzymes, F-box and UBA domains, and deubiquitinating peptidases [20,23,26]. In the eukaryotes there also appears to have been a derivation of at least two domains, namely the POZ and WWE domain through major structural modification of the core β-GF domains.

#### Evolutionary trends in the domain architectures of β-GF domains

**Figure 5:**
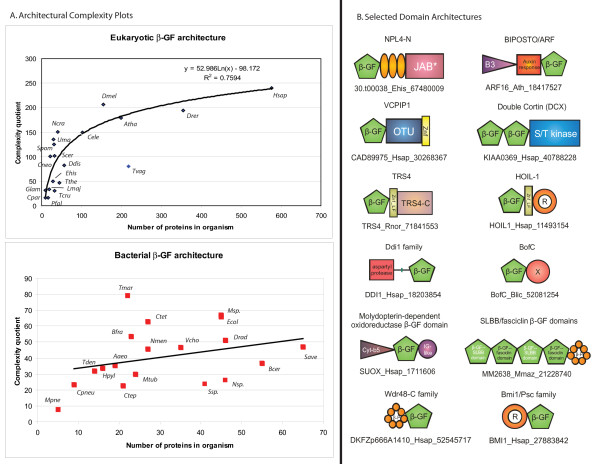
A) Architectural complexity plot of β-grasp domains found in eukaryotes and prokaryotes. The complexity quotient for a given species (y-axis) is plotted against the total number of β-grasp domain containing proteins in the same species. Names of species are given next to plot points. B) Domain architectures of β-grasp domains. Only a small sample of architectures is shown. These mainly represent novel or recently reported architectures that are described in the text. The TRS4 C-terminal domain, also found fused to certain E1-enzymes that lack the C-terminal UFD has a highly conserved ExxxH implying enzymatic function (see Additional file 1 for an alignment). Orange ellipses represent the conserved cysteine clusters observed in the NPL4-N family (see Additional file 1). A straight line with a small green box in the Ddi1 family architecture represents a possible cleavage site located between the domains. The proteins are not drawn to scale as only globular segments are show. Explanation of abbreviations/domain names: B3, DNA-binding domain; Auxin response, auxin-responsive transcription factor domain; OTU, OTU-like family of cysteine proteases; Znf, zinc-finger; Znf_LF, little finger family of zinc finger domains; R, Ring-finger domain; β-P, β-propeller domain; X, previously uncharacterized BofC C-terminal domain also found fused to a serine/threonine phosphatase in actinobacteria (see Additional file 1 for alignment). Organism abbreviations: Ehis, *Entamoeba histolytica*; Ath, *Arabidopsis thaliana*; Hsap, *Homo sapiens*; Rnor, *Rattus norvegicus*; Blic, *Bacillus licheniformis*; Mmaz, *Methanosarcina mazei*; Ddis, *Dictyostelium discoideum*; Lmaj, *Leishmania major*; Tcru, *Trypanosoma cruzi*; Pfal, *Plasmodium falciparum*; Tthe, *Tetrahymena thermophila*; Ncra, *Neurospora crassa*; Drer, *Danio rerio*; Cele, *Caenorhabditis elegans*; Dmel, *Drosophila melonogaster*; Scer, *Saccharomyces cerevisiae*; Tvag, *Trichomonas vaginalis*; Uma, *Ustilago maydis*; Spom, *Schizosaccharomyces pombe*; Cneo, *Cryptococcus neoformans*; Glam, *Giardia lamblia*; Cpar, *Cryptosporidium parva*; Tmar, *Thermotoga maritima*; Mpne, *Mycoplasma pneumoniae*; Ecol, *Escherichia coli*; Vcho, *Vibrio cholerae*; Hpyl, *Helicobacter pylori*; Nmen, *Neisseria meningitides*; Msp., *Mesorhizobium *sp.; Ctet, *Clostridium tetani*; Aaeo, *Aquifex aeolicus*; Tden, *Treponema denticola*; Drad, *Deinococcus radiodurans*; Mtub, *Mycobacterium tuberculosis*; Save, *Streptomyces avermitilis*; Bfra, *Bacteroides fragilis*; Ctep, *Chlorobium tepidum*; Nsp., *Nostoc *sp.; Ssp., *Synecococcus *sp.; Cpneu, *Chlamydophila pneumoniae*.

Previous studies on domains occurring in diverse architectural contexts in multi-domain proteins have hinted at a strong relationship between domain architectures and functional constraints [104]. We systematically analyzed the domain architectures of the β-GF domains and their conservation across evolution to identify these constraints and any role they might have in predicting functions of uncharacterized versions of the domain. Both the sulfur-carrier function and conjugation to other proteins require the free carboxy-terminus of the standalone β-GF domain. As a result, the standalone copies of the 5-stranded UB-like version have been preserved across all three superkingdoms since LUCA. But an alternative strategy to this, observed primarily in eukaryotes, is the generation of free C-termini through post-translational proteolytic cleavage as seen in the polyubiquitins and APG8p (Aut7p). This raises that possibility that there might be other as yet undiscovered versions which are released for conjugation by proteolytic processing, as has been previously proposed for the DWNN domain [86]. In this context, it remains to be seen if the Ub-like domain in the eukaryotic DDI1p-like proteins [39], which is connected via a glycine-rich linker to the rest of the protein (Fig. 5) might be processed by the C-terminal aspartyl peptidase domain release a free UB-like polypeptide.

In contrast, versions involved in protein and nucleic acid interactions are under no major constraints to remain as standalone forms of the domain. Hence, we find numerous instances of β-GF domains involved in this function occurring in multi-domain architectures. The ribosomal proteins tend to be small and usually one or two-domain proteins. Accordingly, there is not much architectural diversity seen in case of forms like L25. The forms found in the DNA-dependent RNA polymerase represent some of the most complex architectures wherein the β-grasp domain is inserted within an RRM-fold domain which in turn is inserted within a larger, multi-domain scaffold [43]. In most cases, the multi-domain architectures of RNA metabolism-related proteins are well-conserved across entire superkingdoms or even the three superkingdoms of Life because of the universality of these functions in their respective phyletic ranges. Multi-domain architectures associated with signaling or small-molecule interactions are often more restricted in their phyletic range and show lineage-specific diversity [105,106]. Consistent with this, considerable lineage-specific diversity is observed in prokaryotic β-GF domains involved in small molecule-binding like the cobalamin-binding SLBB domains and fasciclin domains and certain enzymes such as the molybdopterin-dependent oxidoreductases (Figure 5). All these domains are typically encountered in secreted proteins and form highly variable multi-domain architectures in various bacteria. In some instances two distinct versions of the β-GF domain might occur in the same polypeptide: for example, the fasciclin domain and the molybdopterin-dependent oxidoreductase domains occur in certain secreted enzymes (Figure 5). Conversely, the small molecule-binding β-GF in certain highly conserved intracellular enzymes like glutamine synthetase and aldehyde oxidoreductases do not show much diversity in domain architectures.

To objectively assess the trends in domain architectural complexity, we made use of the previously devised complexity quotient (CQ) [19]. The CQ provides a measure of the complexity of domain architectures in which a given domain occurs (Figure 5). Specifically, it is defined as the product of the number of different types of domains that co-occur with β-grasp domain containing proteins and the average number of domains detected in these proteins. The complexity quotient was plotted against the total number of proteins containing β-GF domains in a given organism. This was done for 19 completely sequenced species of prokaryotes and 19 eukaryotic proteomes spanning the entire currently available phyletic spectrum of organisms with sequenced genomes. In the case of prokaryotes the plot reveals a more or less flat line with an approximately constant domain architectural complexity across all prokaryotes, irrespective of the number of β-GF proteins they possessed (Figure 5). The plot only showed a few anomalous points: there was a greater than expected paucity of β-GF proteins in the highly reduced genome of *Mycoplasma *and an inexplicably high architectural complexity in *Thermotoga maritima*. Thus, barring very few exceptions, the main tendency in prokaryotes is a wide variability in the number of proteins with β-GF domains rather than any concerted increase in architectural complexity.

Eukaryotes not only have greater numbers of β-GF domain proteins, but also appear to display greater diversity of domain architectures relative to the prokaryotes. The complexity of the β-GF proteins as well as their numbers appear to increase throughout eukaryotic evolution with the highest figures observed in multicellular organisms of the eukaryotic crown group. However, the increase in architectural complexity is not linear across eukaryotes, with a tendency to plateau in animals. The only exception to the strong trend is *Trichomonas vaginalis*, a basal eukaryote, which appears to have undergone a massive, relatively recent proliferation across most protein families [107]. As a result it possesses an unexpectedly large number of β-GF proteins, but low architectural complexity comparable to other basal eukaryotes with similar numbers of β-GF-containing proteins (Figure 5). In terms of actual architectures, the multicellular eukaryotes show numerous lineage-specific multi-domain proteins with different β-GF domains, which are often involved in specific signaling pathways that correspond to unique aspects of the biology of these organisms. For example, the programmed cell death pathways in animals and the auxin-response in plants contain representatives with such unique architectures (Figure 5) [19].

Typically, many of the eukaryotic multi-domain architectures, both ancient and lineage-specific, tend to combine the UBL domains with other signaling domains, typically those involved in UB-signaling. These combinations include those with deubiquitinating peptidases (e.g. of the OTU superfamily), E3 ligases usually of the RING superfamily (Figure 5), and other UB-binding domains like UBA, or other kinds of signaling domains like kinases as seen in the IKKs and Doublecortin. Another feature seen in eukaryotic architectures is the architectural variability through domain loss or accretion, even in the case of highly conserved orthologous proteins. For example, the Npl4p family [108] of Ubls is conserved throughout eukaryotes and might play a role as a novel E3 in degradation of proteins in the endoplasmic reticulum. It can be reconstructed as having an ancestral architecture that combined an N-terminal Ubl with a central region containing variable numbers of a novel Zn-chelating cysteine cluster domain and a C-terminal catalytically inactive version of the JAB peptidase domain (Figure 5, see Additional file 1). In the plant lineage the central Zn-chelating cluster is lost, while in animals and fungi an additional Zn-finger domain is inserted N-terminal to the cysteine-rich Zn-cluster.

#### Structural correlates for functional diversity in the β-GF

**Figure 6:**
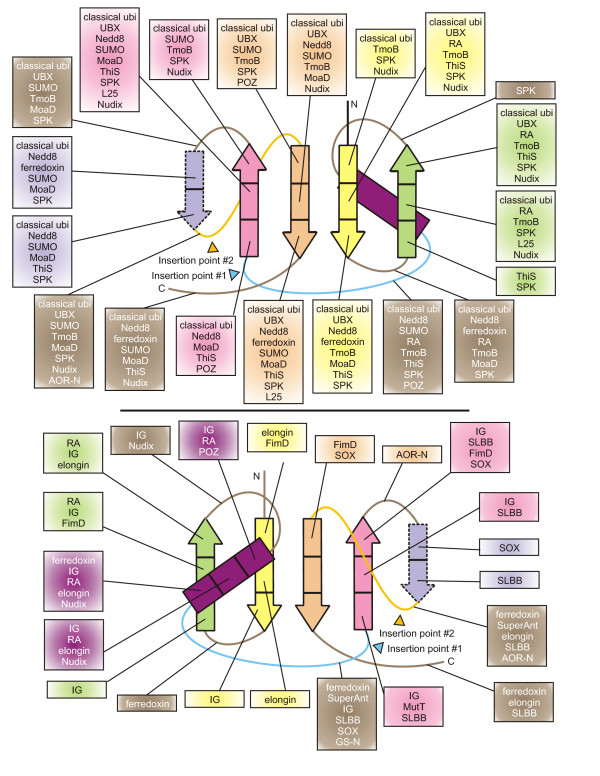
Diagram of relative location of β-grasp interacting partners. The strands and core helix of an idealized β-GF domain have been broken into interaction zones, and the names of representatives of the fold that interact using each of these zones is listed. The top view depicts the exposed face while the bottom view depicts the obscured face. Coloring of the boxes containing lists of specific β-GF domains interacting via a particular region correspond to coloring of structural elements (i.e. a particular strand or loop) involved in the interaction.

We next sought to decipher the relationship between functional diversification and structural elaborations of the fold. For this purpose, we created an idealized representation of the β-GF fold (Figure 6) and divided the structural elements into equivalent zones that are comparable across the available structures. We then mapped interactions to ligands (see materials and methods for details) in all members for which this data is available onto the above scaffold to obtain an interaction map for the fold (Figure 6). We then used this interaction map in conjunction with the above developed classification scheme and relative temporal pattern of diversification to explore the evolution of the structure-function relationships. For the sake of convention, we refer to the exposed surface of the core β-sheet as the "exposed face" and the opposite surface of the sheet which might be obscured by the packing helical segment, the lateral shelf or flange, and the connector arm (in the 5-stranded versions) as the "obscured face". We refer to the C-terminal most portion of the final strand as the "tail".

Little is known of the exact mode of interactions of the basal 4-stranded versions of the fold. However, the apparent rarity of the simple 4-stranded versions suggests that there appears to be a tendency to elaborate the core sheet to provide an increased interface for interactions. On the whole, the exposed face mediates more interactions across the β-GF fold compared to the obscured face. Thus, the proliferation and widespread utilization of the 5-stranded version might be associated with the availability of a larger surface on the exposed face for mediating contacts. Another evolutionary trend is the formation of a barrel-like configuration through insertion of strands which on instances provides a classical interaction interface at the open end of the barrel. We discuss below more specific themes of interaction that were observed in multiple superfamilies of the fold.

##### Solute interaction in the fasciclin-like assemblage

As discussed above, the prevalence of soluble ligands such as sugars, amino acids, and metabolic intermediates for different sequence superfamilies of the clade suggested an ancestral solute-binding role for these proteins. Analysis of the interactions with respect to the shared structural core of this assemblage suggests that the insert and the lateral shelf form an interface for soluble ligand interaction in fasciclin, GS-N, and phosphoribosyl-AMP cyclohydrolase domains [59,77,109]. Furthermore, in glutamine synthetase this interaction might indirectly contribute to catalysis via a conserved aspartate from this region that interacts with the substrate bound at the active site and helps in anchoring it there. This suggests that ancestral versions of this assemblage probably mediated a generic ligand interaction via a similar interface. The interactions of the L25 domain via this interface, if any, remain unknown. However, it is known to contact 5S rRNA via the exposed face [78]. FimD, which appears to be a distant relative of the fasciclin-like assemblage, assumes a classical barrel configuration, with the "open-end" of the barrel providing an interface for interacting with the FimC immunoglobulin domain [61]. Similar "open-ends" of topologically unrelated barrels like the OB fold, PRC, and SH3 barrels are known to mediate interactions with ligands in a like manner [110-112]. The loop between the penultimate two strands of the core FimD β-GF domain is one of the major determinants of the interaction and this feature is comparable to certain interactions of the phosphoribosyl-AMP cyclohydrolase domain (see below).

##### Metal chelation, solute interaction, and prosthetic group attachment in the SLBB/ferredoxin/molybdopterin-dependent oxidoreductase clade

The unifying inserts of this clade typically occur in the region prior to strand 3 and in the region associated with the connector arm or the additional strand of the 5-stranded core. However, there is considerable diversity in the means by which these inserts mediate specific interactions, both between and within different superfamilies of this clade. The 2Fe-2S ferredoxins contain a characteristic set of four cysteines, three of which come from the pre-strand 3 insert and one from the connector arm-associated insert which help in coordination of the 2Fe-2S cluster [113]. The proline dehydrogenase N-terminal domain lineage of this superfamily lacks the cysteines but retains the inserts, suggesting that it might have been reused for interactions with as yet uncharacterized small molecule ligands. As previously shown, members of the SLBB superfamily typified by transcobalamin and related B_12_-binding proteins contain a conserved aromatic residue in the pre-strand 3 insert which plays a central role in binding the ligand [34]. In the molybdopterin-dependent oxidoreductase superfamily, the barrel formed by the pre-strand-3 and the connector arm regions provides an open face for accommodating the molybdopterin ligand. Additionally, a conserved cysteine present in the pre-strand-3 insert is covalently linked to molybdopterin [64]. The above-described evolutionary history of this clade suggests that the ferredoxins were probably the most ancient versions. The subsequent diversification of this clade appears to have involved extensive adaptation of the binding site that originally contained the 2Fe-2S cluster for accommodating a diverse set of new ligands. Additionally, the exposed face in most of these cases remains available for interaction with other domains or polypeptides to recruit the β-GF domain to larger complexes. This has been extensively demonstrated in the case of the 2Fe-2S ferredoxins [114,115].

##### Interactions of AOR-N β-GF domains with metallopterins

The AOR-N domain represents the second independent case of a β-GF domain acquiring the capability to bind tungstoperin or molybdopterin and iron-sulfur clusters (4Fe-4S). Here, a head-to-tail dimer formed by the two tandemly repeated β-GF domains bind metallopterin via the unusually structured connector arms that form a strand at the fringe of the core 5-stranded sheet [65] (Figure 6). While the two tandem repeats are very similar in structure, they are highly divergent in sequence, and contribute different sets of residues in the connector arm to contact the metallopterin. The N-terminal domain contributes an asparagine that directly interacts with the pterin moiety, whereas the C-terminal domain contributes a conserved arginine that interacts with the sulfoxide moiety that chelates the Tungsten or Molybdenum. The same arginine from the C-terminal domain also interacts with the 4Fe-4S metal cluster. Additionally, a threonine from the loop between strand-3 and stand-4 of the core β-grasp domain also interacts with the 4Fe-4S cluster [65] (Figure 6). Thus, the 4-layered sandwich formed by the two derived β-GF domains help in positioning the metallopterin and 4Fe-4S for the C-terminal α-helical domain to catalyze the redox reactions on the substrates. It is possible that this neomorphic mode of substrate interaction arose from MoaD-like precursors that evolved the ability to recognize metallopterins as dimers, as an offshoot of their ancestral function in metallopterin biosynthesis.

##### Principal protein and nucleic acid interactions observed in the 5-stranded assemblage

A diverse range of protein-protein interactions are shown by both prokaryotic and eukaryotic members of the 5-stranded assemblage, including those with the E1, E2 and E3 enzymes or their prokaryotic counterparts. The recently published structure of the complex of Nedd8 with its E1 and E2 enzymes [62], in conjunction with the data accumulated from several other structures and mutagenesis experiments helps in deciphering the key modes of interaction prevalent in the 5-stranded clade. Nedd8 interacts via the exposed face with the sheet of the Rossmann fold domain of the adenylating domain of the E1, as in the case of the ThiS/MoaD clade [48,49]. Similarly the exposed face is also used by the β-GF of the C-terminal UFD of the E1 to recruit the E2. More generally, different parts of the exposed face of the sheet mediate interactions specific to particular representatives of the 5-stranded assemblage (Figure 6). In particular, zones corresponding to the C-termini of the first and last strands which lie in the center of the sheet are utilized for protein interactions by all studied members of the classical 5-stranded clade. The structures of the eukaryotic members of the classical 5-stranded clade show that many of the interaction positions on the exposed face are shared, though the actual residues at those positions might not be conserved. Hence, the interaction specificity of different members has mainly arisen via sequence diversity at spatially congruent interaction sites, as opposed to acquisition of entirely new modes of interaction. The availability of the exposed face that provides an extended surface for interaction appears to be the primary factor for the pervasive use of this fold as mediator of protein-protein interactions across biologically disparate contexts. In a few instances, the obscured face of the RA (PDB: 1LFD [116]) and elongin domains (PDB: 1VCB [117]) might mediate specific interactions suggesting that their adapter function might depend on using both faces to mediate different sets of specific interactions.

In the complex of Nedd8 with its conjugating enzymes, the Nedd8 moiety covalently linked to the cysteine in the thioester-forming α-helical domain of the E1 protein also serves to recruit its specific E2 [62]. This occurs via a unique interaction involving the cleft formed between then sheet and the helix of the β-GF, which constitutes the "open-end" of the barrel-like form of the fold in Nedd8. This is reminiscent of the interaction observed in the FimD-like versions of the β-GF. From the side of the E2, the interaction is mediated via the conserved C-terminal helix. The high diversity of the residues in the E2 helix as well as the cleft of the Ub/Ubls suggests that this interaction is required for the specificity of E2-Ubl association. This interaction is representative of the more generic tendency of peripheral locations on the fold to be deployed in specific interactions that might be required only for the unique function performed by a particular superfamily (Figure 6). In the sulfur carrier and conjugated versions, the C-terminal tail plays a specific role in interaction with the active site of enzymes performing the adenylation or thioesterification [47-49,62]. The role of the exposed face in protein-protein interactions appears to be a conservative aspect of the entire 5-stranded assemblage, which has been preserved from a period predating LUCA. Similarly, the mode of interaction with modifying enzymes via the tail appears to be an ancient conserved one. The apparently complex multiple protein-protein interactions in the eukaryotic Ub-conjugation process also appear to have emerged from the repeated use of the exposed face for interaction with E1, E2 and E3 partners.

In contrast to protein-protein interaction, little is known of the actual mode of RNA-interaction by versions of the classical 5-stranded assemblage like the TGS domain. While it is possible that the exposed face provides an interface as observed in the L25-5S rRNA-interactions [78], the conserved "α-L" structural motif shared by the S4 and TGS domains [69] suggests a role for the obscured face in RNA interactions.

##### Multiple "inventions" of enzymes in the β-GF

At least 3 distinct superfamilies of enzymes use the β-GF the primary scaffold for their active sites. None of the enzymatic forms can be confidently traced to LUCA. Instead they appear to have emerged early in bacterial evolution. Furthermore, the enzymatic versions are only distantly related to each other suggesting that the β-GF fold has been convergently used to provide catalytic scaffolds. In the case of phosphoribosyl-AMP cyclohydrolase and the molybdopterin-dependent oxidoreductases like sulfite oxidase, the main pre-adaptation for catalysis stems from the ancestral ligand binding capabilities that emerged in the assemblages to which they belong. In the case of the Nudix superfamily, the pre-adaptation was possibly the nucleic acid-binding ability of the ancient 4-stranded versions of β-GF. Consistent with the convergent origins of catalysis in each instance emergence of enzymatic activity appears to have resulted in some distinctive structural changes that go beyond the above pre-adaptations shared with ligand-binding forms. In the molybdopterin-dependent oxidoreductases, the structural innovation is in the form of an elaboration of the ancestral ligand-binding features – the extensive inserts resulting in the 3-layered structure with an open barrel-like element provide a pocket for the cysteine-attached co-factor as well as the substrate.

In the phosphoribosyl-AMP cyclohydrolases a sequence alignment and super-position of the conservation onto the available structures indicates two basic active site elements (see Additional file 1). The first of these is a set of conserved residues from the insert and the lateral shelf, which appear to form the basic substrate binding site, as in other characterized members of the fasciclin-like assemblage. The second is the active metal-chelating site formed by 3 conserved cysteines, one from the loop between the last 2 strands of the core fold and two others from a C-terminal β-hairpin extension. Further, these proteins form obligate dimers on account of a strand-swapping interaction between the core β-GF and the C-terminal β-hairpin extension [76]. This juxtaposes the proposed substrate-binding site from one monomer with the metal-chelating site from the other monomer to form two distinct equivalent active sites at the dimer interface. Thus, the emergence of a neomorphic metal-chelating site on the basic fasciclin-like β-GF scaffold appears to have been central to the origin of catalysis in this case.

The primary structural innovation in the Nudix hydrolases is the peculiar "outflow" that juts out of strand-2 on the exposed face (see above). The PXG motif in strand-2 immediately after this "outflow" also provides the space to accommodate substrates due to a lack of large side-chains. Additional substrate contacting positions also come from the rest of the exposed face. However, the conserved residues actually required for catalysis do not come from the exposed face, but from the helical segment and loop connecting strand-2 to it (Figure 6). This loop contains a conserved motif of the form [DE]xxE (where x is any amino acid) and the helix itself contains the motif RExxEE [74,118]. Of these, 4 acidic residues from the two motifs, excluding the last glutamate of the RExxEE motif, form a negatively-charged cloud around the arginine, and appear to be critical for positioning the active site and providing the right polar environment. The last conserved glutamate projects towards the exposed face and directs the attack on the scissile diphosphate linkage [118]. The "outflow" in strand-2 creates the necessary perforation in the sheet that allows the active glutamate to access the scissile bond of the substrate bound on the exposed face. These structural features suggest that in precursors of the Nudix enzymes the β-GF domain most probably bound the substrate via the exposed face, as is common in this fold. The emergence of the "outflow" in strand-2 would have provided further contacts for substrate interaction and at the same time created an aperture in the sheet allowing residues from the helix to form a unique active site.

##### Other atypical modes of interactions

A few modes of interactions thus far appear to be restricted to certain sporadic lineages or are only seen in certain highly derived forms of the domain. The superantigen/toxin-type and strepto/staphylo-kinase-type β-GF domains share an unusual general mode of interaction with plasmin and the T-cell receptor β-chains, respectively. Both appear to contact partners "side-on" via the edge of the domain on which the additional strand of the 5-stranded versions is situated. A comparable "side-on" interaction is also seen in the evolutionarily distant 4-stranded form of the fold found in the Ig-binding domain. However, this latter interaction is distinct in having additional extensive contacts supplied by the helical segment from the obscured face of the domain (Figure 6). It is unclear if this "side-on' interaction might be another less-known but ancient interaction feature of the β-GF domain or has convergently evolved in the superantigen/toxin-type and strepto/staphylo-kinase-type domains on one hand and it the 4-stranded Ig-binding domain on the other. The interactions of the POZ-domain are rather distinct on account of its extreme structural modification as well as acquisition of a unique C-terminal helical sub-domain. However, the structure of the POZ domain in the potassium-channel complexed with the oxido-reductase subunit (PDB: 1EXB [119]) shows that the surface equivalent to the exposed face of the classical β-GF domains plays an important role in the interactions of the POZ domain (Figure 6). This suggests that the POZ domain probably retained at least in part the interaction of the ancestral β-GF domains.

## Discussion and general conclusion

While the β-GF has been thoroughly investigated in the context of the interactions of ubiquitin and UBLs in eukaryotes and their prokaryotic relatives like ThiS and MoaD involved in sulfur transfer, the broader evolutionary history of the fold was poorly understood. We sought to redress this by developing a natural classification for the fold and using it as guide for exploring the tempo of its evolutionary radiations and details of its functional adaptations. As a result we identified several novel members of the fold, including some distinctive previously unidentified modifications. The reconstruction of the evolution of the fold suggests that the major structural variants and some of the basic biochemical features and modes of interaction had emerged prior to LUCA. This suggests that even before the radiation of the extant lineage of Life there were several rounds of duplication followed by extensive divergence, including major structural changes.

The scenario emerging from our analysis also suggests that the earliest reconstructed function of the β-GF domain was in the context of ribonucleoprotein complexes, probably as an RNA-binding domain. Based on the functions of extant versions of the domain, like the TGS superfamily, the IF3-N domain, and early structural derivatives such as the S4 superfamily, it is quite possible that the earliest versions of the fold played a generic role in a primitive pre-LUCA translation system. Thus, the earliest diversification events of the β-GF fold likely occurred in the context of the RNA-world, probably with the acquisition of increasingly specialized roles in the evolving translation apparatus. Amongst the major pre-LUCA functional shifts were those relating to the biosynthesis of sulfur-containing compounds and scaffolding of Fe-S clusters. On the face, such functional shifts from earlier roles in translation-associated RNPs appear drastic and puzzling. However, it should be noted that there is a functional connection between the sulfur incorporation pathways of thiamine biosynthesis and thiouridine synthesis in RNA [88,120]. Hence, it is possible that these shifts might have occurred in the context of 5-stranded versions of the β-GF providing scaffolds for the synthesis of thio-base containing RNAs. This reconstruction also implies that the versions of the β-GF associated with major metabolic functions, including respiratory metabolism, radiated from the ancestral RNA-binding versions.

The major post-LUCA phases of the evolutionary history of the β-GF fold saw two major spurts of innovation. The first, occurring primarily in the bacteria, was accompanied by an extensive exploration of the biochemical function and interaction space by different versions of the fold. This was marked by the acquisition of diverse soluble ligand-binding capabilities through distinctive structural modifications as well as extensive deployment in different protein-protein interaction contexts. Most notably, the scaffold on at least 3 independent occasions acquired very different enzymatic activities even though the β-GF fold did not ancestrally support catalytic activities. The eukaryotic phase did not see extensive innovation in terms of fundamentally different biochemical functions, but the diversity of protein interactions within the ubiquitin-like superfamily of the 5-stranded assemblage was vastly expanded through extensive sequence divergence of the primary interaction surfaces of the superfamily. This phase was also accompanied by ongoing innovation of new multi-domain architectures associated with the eukaryotic expansions of Ub-like signaling domains (Figure 5).

As has been suggested before, the β-GF shows several parallels with the RRM-like fold [34]. Both are relatively small folds, forming asymmetric 2-layered structures, with one face of their sheets exposed, and the other partially or wholly obscured by helical segments. Importantly, when centered on the β-hairpin element in core of their sheets, strands in both these domains and one helix show the same orientation. Both have evolved to provide scaffolds for a comparable set of diverse biochemical functions, including RNA-binding, small-molecule or solute recognition, protein-binding, supporting Fe-S clusters (ferredoxins) and providing the skeleton for active sites of very different enzymes. Both of these folds also appear to have undergone extensive adaptive radiation even prior to LUCA after starting off as domains with primitive roles related to RNA metabolism [121]. These two folds differ from the ancient α/β3-layered sandwich domains like the Rossmannoid and P-loop NTPase domains, which appear to have begun their existence as enzymatic domains and more-or-less retained a conserved set of basic biochemical activities throughout their evolution [121,122]. The versatility of the β-GF and RRM-like folds in providing scaffolds for both enzymatic and diverse non-enzymatic function might be attributable in part to two major factors: 1) an issue of contingency – these folds simply arose very early in evolution and had the time to colonize numerous functional roles. 2) Favorable structures – their relatively large sheet that is exposed on one side provides an interface for diverse interactions, especially in the form of binding various substrates. Sequence alteration to this binding surface, without disrupting the overall scaffold, could easily allow the emergence of a great diversity of new interactions. Secondly, the presence of the large sheet with just a single helical segment also favors formation of barrel-like structures, thereby opening new faces for interactions.

Despite intense investigations the precise functions of several eukaryotic Ubls remain unclear. More generally, the functional details of the non-ubiquitin-like members of the fold remain less studied, as in the case of the β-GF domains in RNP complexes which are in need of more detailed investigations. In conclusion, we hope that our analysis of the β-GF domain provides a new framework for further systematic experimental exploration of the functions of this fold.

## Methods

The non-redundant (NR) database of protein sequences (National Center for Biotechnology Information, NIH, Bethesda, MD) was searched with the BLASTP program [52]. Profile searches were conducted using the PSI-BLAST program [52] with either single sequences or multiple alignments as queries, with a profile inclusion expectation (e) value threshold of 0.01; searched were iterated until convergence. Hidden Markov models (HMMs) built from alignments using the hmmbuild program were also employed in searches carried out using the hmmsearch program from the HMMER package [123]. For queries and searches containing compositionally biased segments, the statistical correction option built into the BLAST program was used [124]. Multiple alignments were constructed using the MUSCLE [125] and/or T-COFFEE programs [126], followed by manual adjustment based on PSI-BLAST hsp results and information provided by solved three-dimensional structures. All large-scale sequence and structure analysis procedures were carried out with the TASS software package (V. Anantharaman, SB and LA, unpublished results), a successor to the SEALS package [127]. Protein structures were visualized using the Swiss-PDB viewer [128] and cartoons were constructed with the PyMOL program [129]. Protein secondary structure predictions were made with the JPRED program [53], using multiple alignments as queries. Phylogenetic analysis was carried out using a variety of methods including maximum-likelihood, neighbor-joining, and minimum evolution (least squares) methods. Maximum-likelihood distance matrices were constructed using the TreePuzzle 5 program [130] and were used as input for the construction of neighbor-joining with the Weighbor program [131]. Additionally, trees were constructed using the neighbor-joining and minimum evolution methods as implemented in the MEGA program [132] and the Bayesian inference method using Markov chain Monte Carlo simulations implemented by the MRBAYES program [133].

Structure similarity searches were conducted using the standalone version of the DALI program [54,55] with the query structures scanned against local current version PDB that has all chains as separate entries. The structural hits for each query was collected, even if the DALI Z-score for the match was less than 2.0 and parsed for topological congruence to the β-GF template (Table 1) using a custom PERL script. To assess topologically congruence, coordinates of the matching regions detected by DALI searches using known β-GF domains as queries were extracted and analyzed for secondary structure using DSSP program. These secondary structure elements were then represented as a string (corresponding to a row in table 1) along with the polarity of the secondary structure element determined from the DALI match to the query structure. These strings were then matched with the equivalent secondary structure pattern strings constructed of *bona fide *β-grasp domains. If a complete match was obtained these structures were tagged as congruent, while those which were not were ranked in descending order of elements that did not match. This discrimination of the potential candidates was further confirmed by visual examination of each structure. The interacting residues of various proteins of β-grasp fold with their interacting molecules have been deduced using custom made PERL scripts. The scripts encode interacting distance cut-off values of 5.0 Å and 3.5 Å between appropriate atoms in 3-D for deducing the hydrophobic and polar interactions respectively. These inferred interactions were further examined manually using Swiss-PDB viewer for confirming the contacts between amino-acid residues of β-grasp fold proteins and atomic groups of interacting partners.

## Reviewer's report 1

Igor B. Zhulin, Oak Ridge National Laboratory and University of Tennessee, USA

The current study is related to the previous work by the authors reporting a novel domain superfamily within the β-grasp fold (*Biology Direct *2007, **2**:4). In this investigation, authors launched a large-scale exploration of the β-grasp fold diversity and evolution. This is an important, but daunting task, because the fold is well populated and comprises divergent domain families. In my opinion, both strengths and weaknesses of the paper are resulting from this fact.

### Strengths

The main strength of this paper is systematic and logical classification of the fold, defining its core elements and linking numerous elaborations to functional diversity. This is well done by using an array of computational techniques interrogating both sequence and structure. Natural classification of protein folds and domains is urgently needed, thus this work makes a significant contribution to the field. An important element of this study is uncovering several novel domain groups. For example, I was intrigued by the finding that flagellar hook-filament junction proteins FlgL and FlgK are related to the flagellar cap protein FliD comprising a new version of the 5-stranded β-GF clade, Spatial distribution and function of these proteins in the flagellar assembly are quite similar; however, their relatedness in sequence or structure has never been demonstrated before. I am certain that other facts unearthed in this work may draw attention of other specialists studying various systems involving β-GF proteins.

### Weakness

In my opinion, the weakness of this manuscript is in its organization. Potentially, this can be improved, although I keep the door open for a counterargument by not providing constructive suggestions on how to do that. Because there is such a large body of information, its organization is important. As for any scientific contribution, it is imperative to identify what is the status quo in the field and what has been done by the investigators to advance our knowledge. This particular paper is written as mix of a thorough literature review (there are 162 references!) and an original study. The purpose of the former is (hopefully) to synthesize novel ideas/leads from numerous known facts, whereas the purpose of the latter is to generate novel facts filling the gaps in knowledge. This paper does both, but it is does not *effectively *separates the two. If one wants to know exactly which facts are newly discovered (and there are many of them!), it will take some time... Ideally, all information pertinent to the known facts should be introduced in the Introduction and Discussion, whereas Results should show clearly what new information has been obtained in this study. I think combining Results and Discussion in one section creates this feeling that known and novel facts are mashed together. I know that it is much easier for authors to present such a complex study in this way, but the trade-off is the lack of above mentioned distinction. One suggestion on improving the overall organization of the current scheme is to introduce numerical headings and subheadings. For example, in the current version of the manuscript the reader is left to wonder whether "Basal versions of the β-GF" is a subheading of "Natural classification of domains" or they are equal headings, and so on. Having 1., 1.1, 1.1.1. system should help to see the logic of the story better.

#### Author response

*Given the intricate connection between the results and inferences drawn from them, we felt it better to combine the Results and Discussion sections to avoid redundancy. We have now reworded the text to more clearly differentiate the discoveries presented in our work from those that are already known. Accordingly, we have also revised the heading style and added subheadings, where appropriate to improve clarity. While we could have used a numbered style for greater clarity as recommended by the referee this scheme does not fit into Biology Direct's manuscript style*.

### Neutral feeling

Essentially half of the manuscript is devoted to developing a scenario for the fold evolution. This scenario is certainly plausible, as there are no obvious flaws or contradictions. One the other hand, the starting point of inferring relationships is structural information and so is the end point of their verification. I might be wrong here, but this is the feeling. I wish we would have an independent method (or better methods) of testing these relationships more rigorously.

#### Author response

*Given the small size of the domain, the absence of catalytic, active site residues and the level of sequence divergence several superfamilies of the β-grasp fold can only be unified by structural features. As a result, shared structural features are a principal guide for developing the evolutionary at a higher level scenario. However, as mentioned in the text, we also used distance trees based on pairwise Z-scores of structural comparisons to infer evolutionary relationships between different superfamilies. Our final classification is thus a parsimonious reconstruction that includes information obtained in both these methods. Thus, what is presented is a phylogenetic hypothesis that is amenable to future falsification. The utility of this phylogenetic hypothesis is in developing a framework for exploring emergence of various properties of different superfamilies of the fold. We do appreciate the referee's concern for the means for independent falsification of the presented hypothesis. We believe these do exist even if not straight forward: 1) Availability of new structures can allow testing of the suggested scenarios to present evidence for and against the scenario presented here. 2) The scenario presented here makes predictions of certain functional properties such as binding interfaces and substrates that explicitly stem from the phylogenetic analysis. Testing these predictions allows a test of the underlying scenario*.

### Specific comments

2. On page 6 there is a reference to HMM searches initiated with models built from multiple alignments; however, there is no mentioning of the HMMER package in the Materials and Methods.

#### Author response

*We have added a brief description of HMM searches to Materials and Methods*.

## Reviewer's report 2

Arcady Mushegian, Stowers Institute for Medical Research, Kansas City, USA

Throughout the paper, consider changing "hits" to "matches" in the context of BLAST searches: "hit" used to have a special technical meaning, e.g. "using the two-hit method to trigger ungapped extensions".

### Author response

*We admit this possibility of potential misunderstanding and make the suggested change*.

Pg 5 of the pdf, 4th ln from the bottom: what is "topological similarity search"? It is not defined in M&M.

Pg 6, par 2, ln 8. What is "congruence to the unique topological template", how such congruence was evaluated, and what if something is "incongruent"?

### Author response

*We have added a description of this procedure to the Materials and Methods. We now describe how congruence was evaluated*.

Pg 7 ln 5–6. Conserved pattern of hydrophobic interactions in the core is said to strongly support its monophyletic origin. I am wondering what is the null hypothesis and the burden of proof here: surely, there are also some weakly conserved and non-conserved hydrophobic interactions – are there more of them than the conserved ones? If so, do they reject the hypothesis of the monophyletic origin? Is there any test that can reject such a hypothesis for proteins with a shared fold?

### Author response

*With respect to ancient folds (which were already definitely present in LUCA, like β-GF) there are two main scenarios, both of which based on our current understanding are possible: 1) Monogenesis of all ancient folds, or at least major subsets of them, like the α+β two layered fold category to which the β-GF, RRM, ASCR etc belong.2) Polygenesis in which various distinct α+β two layered folds have independently arisen and convergently adopted similar general structural features. If the former were the case, then the hypothesis of domains not sharing a fold cannot be rejected in an absolute sense. However, one could still define exclusive monophyletic groups*.*Under both scenarios we believe that the null hypothesis would be that non-monophyly (or exclusive monophyly) of two or more distinct domains with the burden of proof being the need provide evidence for their exclusive monophyly. Under both scenarios an exclusive monophyletic group or a distinct fold would have a unique conserved network of hydrophobic interactions. This concept is an extension of a monophyletic protein superfamily defined based on sequence similarity: the superfamily has a distinctive constellation of conserved set of residues – similarly, there are distinct conserved networks of hydrophobic residues that are present in the β-GF or the RRM fold. Just as in the sequence alignment the presence of many non-conserved positions does not reject the monophyly of the aligned sequences, there being many non-conserved interactions in the hydrophobic network does not reject the monophyly of domains with a shared fold. However, the absence of the conserved network characteristic of a fold in domain casts doubt on its belonging to the fold. This is especially so, if there are notable structural differences and a hydrophobic network that corresponds to some other fold. For example the BtrG-like proteins (1vkb A) and the tRNA ribosyltransferase-isomerase 1vky B show some general similarity with the β-GF. However, they lack the conserved hydrophobic core shared by the β-GF. The former has instead emerged from an internal repeat of simpler units and the later might be interpreted as distinct barrel with different stabilizing interactions. Hence with current data these are rejected as members of the β-grasp fold*.

Pg 12 par 3: "The strong conservation of this unique structural feature as well as the exclusive grouping of these versions in structure similarity-based clustering": are those indeed two independent lines of evidence, or is the latter a direct consequence of the former?

### Author response

*We did not imply anywhere that these two are entirely independent forms of evidence. We agree that the presence of a shared strand does contribute to increasing the structural similarity of the 5-stranded group. However, their clustering based structural similarity Z-scores does not exclusively depend on the presence of that additional strand alone. There are other more subtle, or "distributed" elements of similarity, which contribute to their exclusive clustering. In an experiment in which a representative was chosen from each lineage in figure *3*, the strand 5 left out, and then clustered using the MUSTANG program we still recover their exclusive grouping supporting the above-stated contention*.

Pg 15 par 2: What is "principle of phylogenetic bracketing"? If it is the same as "Extant Phylogenetic Bracket" attributed to L. Witmer, then this is not a suitable method for inferring ancestral function, in particular when it is distributed sporadically in the extant lineages.

### Author response

*Witmer's extant phylogenetic bracketing is used to infer a feature of an extinct clade which is bracketed by the features in two extant clades with the underlying assumption of the parsimony principle. We do not imply the above on page 15, but admit that our usage of the term would result in confusion with the above. Accordingly, we modify the discussion in this regard to avoid using the potentially confusing term "phylogenetic bracketing"*.

Text and Fig. 3: it is perhaps unadvisable to call POZ, IGB and BofC "basal versions"

### Author response

*The reviewer is absolutely correct, we in no way intended to refer to these lineages as basal versions, but rather sporadic versions potentially derived from more widely distributed 4-stranded versions. We have altered to text and the label in Fig. *3*to represent this point more accurately*.

Pg 31 ln 6 of M&M: it is "compositionally biased" not "computationally biased".

### Author response

*We have made the correction*.

## Reviewer's report 3

Frank Eisenhaber, Institute of Molecular Pathology, Vienna, Austria

The work by Burroughs et al. is a careful, virtually encyclopedically complete collection of β-GF protein segments in sequence databases and a meticulous analysis of the associated structural and functional knowledge in the literature. For the reviewer, it was especially interesting to follow the discussion of the authors how the splitting of evolutionary lines was associated with critical evolutionary events (pre- and post-LUCA distinction, emergence of kingdoms) and how the functional capabilities of the fold got realized during history of life and how they are associated with elaborations of the basic fold.

The value of this work for the annotation of uncharacterized sequences could be enhanced if the authors made some of their results publicly available in electronic form (for example, protein segments annotated with the classification from this work or subfamily alignments) as supplement to this paper.

### Author response

*Within the provided additional file is a super alignment including many major families within the classical ubiquitin superfamily, as well as individual alignments of all newly-discovered β-GF domains. Additionally, we have provided the protein identifier (gi) numbers along with a brief description of all sequences (including organism name and lineage in which the sequence is found) for all identified families of the β-GF*.

#### Minor issues

1) last sentence of last paragraph of section "Identification of β-GF domains"

Some quantitative data illustrating the comparisons of distributions of previously characterized domains would be desirable. The statement that the majority of conserved lineages has been determined is very strong and depends on the upper level of allowed sequence and structural deviation.

### Author response

*We now write, "a significant fraction" instead of majority. We have also referred to a previous work on the matter*.

2) The reader might be offended to find the definition of domain architectural complexity only in the figure legend of Fig. 5. It would be better placed into the main text.

### Author response

*We have moved the definition of the complexity quotient from the figure legend to the main text*.

## Supplementary Material

Additional file 1 β-grasp families: members, phyletic patterns, and selected alignments. A mega-alignment of several prokaryotic and eukaryotic β-GF lineages and alignments of all newly-identified β-grasp folds and novel ancillary domains are provided along with gi numbers of the sequences collected from iterative database searches and the phyletic pattern of each β-grasp family. Click here for file

Additional file 2 Natural classification of the β-GF. Superfamilies/families/subfamilies of the β-GF are grouped according to shared structural and sequence features, indentations represent the inferred hierarchy of evolutionary relationships based on these features. Major shared features of a set of superfamilies are given between dashed lines and labeled with roman numerals. Superfamilies are written in bold text, with families and subfamilies written in bold, italicized text. Phyletic distribution of a family/subfamily are given in parentheses after name. Brief description of functional role is given following phyletic distribution where known. Selected PDB identifiers of solved crystal structures are indented and listed underneath family/superfamily. Click here for file
