# Supplementary material for: Small but versatile: the extraordinary functional and structural diversity of the β-grasp fold
Source: Biol Direct. 2007 Jul 2;2:18. doi: 10.1186/1745-6150-2-18 (PMC1949818; doi:10.1186/1745-6150-2-18)
Supplement: Additional file 2 — Natural classification of the β-GF. Superfamilies/families/subfamilies of the β-GF are grouped according to shared structural and sequence features, indentations represent the inferred hierarchy of evolutionary relationships based on these features. Major shared features of a set of superfamilies are given between dashed lines and labeled with roman numerals. Superfamilies are written in bold text, with families and subfamilies written in bold, italicized text. Phyletic distribution of a family/subfamily are given in parentheses after name. Brief description of functional role is given following phyletic distribution where known. Selected PDB identifiers of solved crystal structures are indented and listed underneath family/superfamily. [file 1745-6150-2-18-S2.pdf]

Additional file 2. Natural classification of the β-GF.

I. BASAL 4-STRANDED VERSIONS OF THE β-GF

A. Archaeo-eukaryotic RNA polymerase β-subunit domain superfamily (all eukaryotes, all archaea) function unknown
PDB: 1I6HB (residues ~573-631)

B. IF3-N terminal domain superfamily (all bacteria, all eukaryotes except *Giardia*) function unknown
PDB: 1TIF (residues ~9-61)

C. **POZ superfamily** (all eukaryotes) mediates dimerization and transcriptional repression and interacts with histone deacetylase co-repressor complexes
PDB: 1HV2, several others

D. BofC/IGB lineage

** ***Bypass of forespore (Bof)C family*** (*Bacillus*, *Geobacillus*) secreted protein important in spore formation pathway
PDB: 2BW2

Immunoglobulin-binding (Ig-binding) family (firmicutes) cell surface virulence protein
PDB: 1HEZ, several others

E. Other lineages

*****Yml108w family*** (budding yeast) function unknown
PDB: 1N6Z

II. Nudix (MutT) Superfamily

(all eukaryotes, crenarchaea, euryarchaea, all bacteria, dsDNA viruses) nucleotide-derivative phosphohydrolase, contains insert in middle of second strand
PDB: 1RYAA, several others

III. Fasciclin-like assemblage

A. Glutamine synthetase N-terminal domain (GS-N) superfamily (all eukaryotes except *Giardia*, crenarchaea, euryarchaea, all bacteria, mimivirus) two-stranded insert contributes residue to enzyme active site, forms one wall in active site
PDB: 2BVCA (residues ~17-100), several others

B. **Phosphoribosyl AMP cyclohydrolase (HisI) superfamily** (fungi, plants, crenarchaea, euryarchaea, all bacteria) Two enzyme active sites formed by an obligate HisI dimer
PDB: 1ZPS

C. **Fasciclin I superfamily** (apicomplexa, crown group, euryarchaea, actinobacteria, bacteroidetes, chlorobi, chlamydiae, chloroflexi, cyanobacteria, deinococci, acidobacteria, planctomycetes, α/β/δ/γ proteobacteria) binds sugar moieties
PDB: 1NYO, 1O70

D. **Ribosomal protein L25 superfamily** (apicomplexa, plants, slime molds, *Anopheles*, all bacteria) binds 5S rRNA
PDB: 1DFU, several others

E. **FimD N-terminal domain (FimD-N) superfamily** (α/β/δ/γ proteobacteria, deinococci) interacts with FimC protein
PDB: 1ZE3, 1ZDV, 1ZDX

F. WWE superfamily (all eukaryotes except *Giardia*)
PDB: 2A90A

IV. 5-stranded assemblage: Classical 5-stranded clade

A. TGS superfamily (all eukaryotes, all archaea, all bacteria)
PDB: 1NYR (residues 1-62), several others

B. ThiS superfamily (crenarchaea, euryarchaea, all bacteria, algae, *Thalassiosira*, *Emiliania*, *Phaeodactylum*, *Odontella*)
PDB: 1F0Z, many others

C. MoaD superfamily (crenarchaea, euryarchaea, all bacteria, crown group)
PDB: 1FM0, many others

D. Urm-1 superfamily (all eukaryotes) conjugated version implicated in budding and nutrient sensing [1]
PDB: 1XO3, 1WGK, 2AX5

E. **Ribosomal protein S4 superfamily** (all eukaryotes, all archaea, all bacteria)
PDB: 2CQJA

F. **Aldehyde oxidoreductase N-terminal (AOR-N) domain superfamily** (crenarchaea, euryarchaea, actinobacteria, chloroflexi, deinococci, firmicutes, α/β/δ/ε/γ proteobacteria) tandem repeats in same protein form dimer that recognizes metallopterins
PDB: 1AOR (residues 1-210)

G. Prokaryotic UB-like superfamily (including Mut7-C fusion family) phyletic patterns previously described in detail [2]

H. Eukaryotic UB-like superfamily (see section VI. below)

I. SPK/SupAnt lineage

Strepto/staphylococcus kinase (SPK) family (*Streptococcus*, *Staphylococcus*, caudovirales) interacts with host plasmin protein, promoting virulence
PDB: 2SAK, several others
Superantigen (SupAnt) family (*Staphylococcus*, *Streptococcus*) interacts with T-cell receptor β-chains
PDB: 1TY0 (residues 104-211), several others

J. Other lineages of note
TmoB family (actinobacteria, α/β/γ proteobacteria)
PDB: 1T0Q, 1T0R, 1T0S
YukD family (actinobacteria, firmicutes)
PDB: 2BPS
RnfH family (α/β/γ proteobacteria)
*****FliD-FlgL/K family*** (α/β/δ/ε proteobacteria, clostridia, planctomycetes, *Thermotoga*, spirochetes, acidobacteria, Mu-like phages)

V. 5-stranded assemblage: Soluble ligand binding or metal chelating clade
----
A. 2Fe-2S ferredoxin superfamily
2Fe-2S family (all eukaryotes, all archaea, all bacteria) small insert with conserved cysteines chelates Fe ions
PDB: 1NEK (residues 1-106), several others
*****L-proline dehydrogenase-type oxidoreductase (L-proDH alpha) family*** (euryarchaea, *Thermofilum*, actinobacteria, firmicutes, *Psychroflexus*, *Herpetosiphon*, α/β/δ/γ proteobacteria) lack conserved cysteine residues
PDB: 1Y56A (residues ~10-94)

B. ****Molybdopterin-dependent oxidoreductase (SOX) superfamily** (*Toxoplasma*, crown group, crenarchaea, euryarchaea, actinobacteria, *Aquifex*, bacteroidetes/chlorobi, chloroflexi, cyanobacteria, deinoccoci, acidobacteria, firmicutes, planctomycetes, α/β/δ/ε proteobacteria) two inserts in core β-sheet help facilitate enzymatic activity
PDB: 1SOX (residues ~150-310)

C. Soluble-ligand binding β-grasp (SLBB) superfamily
Nqo1 family (kinetoplastids, ciliates, crown group, euryarchaea, all bacteria)
PDB: 2FUGS (residues ~246-334)

Transcobalamin family (hexapoda, vertebrates, euryarchaea, firmicutes, planctomycetes)
PDB: 2BBC (residues ~330-415)

VI. Eukaryote UB-like superfamily members

A. Classic UB-like clade
NIP45/Reni family (crown group)
SUMO/SMT3 family (all eukaryotes) conjugated versions tag proteins for localization/regulation
PDB: 1TGZ, several others
UFD of E1 C-terminal UB-like domain family (all eukaryotes) C-terminal adaptor domain of E1-like enzymes that bind E2-like enzymes [3] [4]
PDB: 1Y8X
UBX family (all eukaryotes except *Giardia*) adaptor version regulating degradation at ER [5] [6]
PDB: 1H8C, several others
Rad23N family (all eukaryotes except *Giardia*) involved in protein recruitment to the proteosome [7]
PDB : 1UEL, 1P1A
Sin3a/SAP18-Ddi1 family
Sin3a/SAP18 subfamily (*Trichomonas*, apicomplexa, ciliates, crown group)
PDB: 2HDE
DNA damage inducible 1 (Ddi1) subfamily (plants, fungi, animals) adaptor version regulating Ho interaction with proteosome [8] [9]
PDB: 1V5O
Apg8-Apg12-APG5 family
Apg8 subfamily (*Trichomonas*, *Naegleria*, kinetoplastids, apicomplexa, ciliates, *Phytophthora*, crown group) target lipids to autophagy pathway [10]
Apg12 subfamily (*Trichomonas*, *Naegleria*, ciliates, crown group) targets proteins to autophagy pathway [10]
PDB: 1WZ3
APG5 subfamily (*Trichomonas* kinetoplastids, apicomplexa, ciliates, entamoebidae, crown group)
PDB: 2DYM
*****Bmi1/Psc-Wdr48C family***
*****Bmi1/Psc subfamily*** (apicomplexa, ciliates, plants, animals)
*****Wdr48 C-terminal UB-domain subfamily*** (crown group)
BIPOSTO/ARF-PB1 family
*****BIPOSTO/ARF subfamily*** (plants) plant transcription factor

PB1 subfamily (all eukaryotes except *Giardia* and *Trichomonas*) adaptor that regulates localization [11]
PDB: 2BKF, several others
MUBs family (plants, fungi, animals) versions anchored to plasma membrane via prenylation [12]
PDB: 1WHG, 1SEH9
Nedd8 family [13] [14] (crown group)
PDB: 1NDD, others
BAG N-terminal domain family (plants, animals) adaptor version mediating proteosome and Hsc70/Hsp70 chaperone system interaction [15]
PDB: 1WXV
Splicing factor 3a (Sf3a)/prp21 family (plants, animals)
PDB: 1WE7, 1ZKH
** ***UBP11/Usp40N-GGNB1 family***
****Usp40 N-terminal UB domain subfamily** (ciliates, slime molds, animals)
****UBP11-GGNB1 UB domain subfamily** (plants, slime molds, vertebrates)
Hepatocyte odd protein shuttling (HOPSP) family (animals)
PDB: 1WIA
Parkin family (entamoebidae, animals) adaptor that binds Rpn10 subunit of 26S proteosomal subunit [16]
PDB: 1IYF, 1MG8
S30 N-terminal fusion ribosomal protein (S30-N) family (animals) adaptor that associates with Bcl-G and histone 2A [17]
Midnolin family (animals) regulates genes related to neurogenesis in the nucleolus [18]
Bone marrow stromal cell-derived (BMSC) UB family (animals) may regulate BMSC function in cell differentiation [19]
PDB: 1X1M
Dendritic cell-derived C-terminal (DC-C) UB domain family (animals) implicated in cell differentiation and apoptosis [20]
****TRS4 N-terminal domain UB family** (animals)
PDB: 2DAF
IkappaB kinase beta (IKK) UB-like domain family (animals) domain required for kinase activity [21]
GDX N-terminal domain family (fungi, animals)
Homocysteine-inducible, endoplasmic reticulum stress-inducible protein (Herp-1) UB domain family (animals)
AN1 UB-like domain family (animals)

Interferon-inducible protein (ISG-15) UB domain family (vertebrates) conjugated version that tags proteins as part of antiviral response pathway [22]
PDB: 1Z2M
2'-5' oligoadenylate synthetase-like protein C-terminal (Oasl2-C) UB domain family (vertebrates) interacts with MBD1 transcriptional repressor [23]
PDB: 1WH3
Classic UB family (all eukaryotes) conjugated versions that target a protein to a cellular destination
PDB: 1XD3, many others
Sacsin UB-domain family (vertebrates)
****Rb1cc1 family** (animals)
FAT10/Diubiquitin family (slime molds, vertebrates) conjugated version that tags proteins for proteosomal degradation [24]
Np95-like ring finger protein N-terminal (NIRF-N) UB domain family (vertebrates)
PDB: 1WY8, 2FAZ
HOIL-1 UB-like domain family (animals) adaptor protein that regulates degradation of suppressor of cytokine signaling (SOCS) proteins [25]
Transcription elongation factor B (Elongin B) family (animals) positive regulator of RNA pol II elongation factor A [26] [27], possible tumor and cytokine signaling complex suppressor [28] [29], and hypoxia-inducible gene regulator [30]
PDB: 1VCB, several others
Nedd8 ultimate buster-1 (NUB1L) N-terminal UB domain family (animals) adaptor version linking FAT10 with the 26S proteosome [31]
PDB: 1WJU
Ubiquilin N-terminal UB domain family (animals) adaptor version that interacts with PDI and mediates delivery of tagged proteins to the proteosome [32]
USP48/USP26 C terminal UB domain family (plants, animals)
U11/U12 snRNP 25K family (plants, animals)
PDB: 1V2Y
Glycoprotein, synaptic 2 N-terminal (Gpsn2-N) domain family (apicomplexa, plants, slime molds, animals)
Ubl5-HubA family
Ubl5 subfamily (*Trichomonas*, apicomplexa, crown group) possibly a conjugating version, although target unknown [33]

HubA subfamily (*Tetrahymena*, fungi) appears to modify Snu66 in pre-mRNA splicing and localization [34], conjugates with cell polarity factors Sph1 and Hbt1 [35]

TUG-UBL1 N-terminal UB domain family (*Naegleria*, slime molds, fungi, animals)

PDB: 2AL3

****CLU1/eIF-3 family*** (*Naegleria*, ciliates, crown group)

VCPIP1- HIP7PN family

Valosin-containing protein (p97)/p47 complex-interacting protein p135 (VCPIP1) UB domain subfamily (vertebrates)

*****HIV-induced protein-7-like protease N-terminal (HIP7P-N) UB-like domain subfamily*** (all eukaryotes except Giardia)

UBP7/UBP14N-Ublcp1-Atg35690N family

UBP7/UBP14 N-terminal UB domain subfamily (all eukaryotes except *Giardia*) likely adaptor protein binding to p53 [36] [37]

PDB: 1V86, 1WGG

Ublcp1 UB domain subfamily (crown group)

Atg35690 N-terminal UB domain subfamily (fungi, plants)

Bat3/DsK family (all eukaryotes) adaptor version that binds with Hsp70-like Stch [38]

PDB: 1WX9, 2BWF, 2BWE

*****AT23465p C-terminal domain family*** (*Trichomonas*, *Giardia*, kinetoplastids, ciliates, animals) fused to cytochrome b5-like heme/steroid binding domain

TbcB-TbcE family

Tubulin binding cofactor B (TbcB) UB-like domain subfamily (all eukaryotes except *Trichomonas*)

PDB: 1T0Y, 1V6E

Tubulin binding cofactor E (TbcE) UB-like domain subfamily (kinetoplastids, ciliates, plants, animals)

PDB: 1WJN

B. RA/FERM/PI3KN/DWNN clade

Ras-associating (RA) family (slime molds, fungi, animals)

PDB: 1RAX, several others

FERM UB-like domain family (ciliates, plants, slime molds, entamoebidae, animals)

PDB: 1EF1 (residues 4-87), several others

Phosphoinositide 3-kinase N-terminal (PI3K-N) domain family (all eukaryotes)

PDB: 1E8Y (residues ~217-310), several others

DWNN (RBBP6 N-terminal domain) family (all eukaryotes except *Giardia* and kinetoplastids)

PDB: 27CH

C. CAD/DCX clade

CAD domain family (animals) adaptor version that inhibits DNAse activity

PDB: 1IBX, several others

Double cortin (DCX) domain family (all eukaryotes except *Giardia*) occur in tandem, function as microtubule-binding domains [39]

D. Additional 5-stranded classical UB-like lineages

BM-002/Ufm1 family (all eukaryotes except *Trichomonas*) conjugated version [40]

PDB: 1J0G

NPL4 N-terminal (NPL4-N) UB-like domain family (all eukaryotes)

PDB: 1WF9

Footnotes

**: β-GF domain described for the first time in this work

REFERENCES

1. Goehring AS, Rivers DM, Sprague GF, Jr.: **Attachment of the ubiquitin-related protein Urm1p to the antioxidant protein Ahp1p.** *Eukaryot Cell* 2003, **2:**930-936.
2. Iyer LM, Burroughs AM, Aravind L: **The prokaryotic antecedents of the ubiquitin-signaling system and the early evolution of ubiquitin-like beta-grasp domains.** *Genome Biol* 2006, **7:**R60.
3. Huang DT, Paydar A, Zhuang M, Waddell MB, Holton JM, Schulman BA: **Structural basis for recruitment of Ubc12 by an E2 binding domain in NEDD8's E1.** *Mol Cell* 2005, **17:**341-350.
4. Huang DT, Hunt HW, Zhuang M, Ohi MD, Holton JM, Schulman BA: **Basis for a ubiquitin-like protein thioester switch toggling E1-E2 affinity.** *Nature* 2007, **445:**394-398.
5. Neuber O, Jarosch E, Volkwein C, Walter J, Sommer T: **Ubx2 links the Cdc48 complex to ER-associated protein degradation.** *Nat Cell Biol* 2005, **7:**993-998.
6. Schuberth C, Buchberger A: **Membrane-bound Ubx2 recruits Cdc48 to ubiquitin ligases and their substrates to ensure efficient ER-associated protein degradation.** *Nat Cell Biol* 2005, **7:**999-1006.
7. Madura K: **Rad23 and Rpn10: perennial wallflowers join the melee.** *Trends Biochem Sci* 2004, **29:**637-640.
8. Ivantsiv Y, Kaplun L, Tzirkin-Goldin R, Shabek N, Raveh D: **Unique role for the UbL-UbA protein Ddi1 in turnover of SCFUfo1 complexes.** *Mol Cell Biol* 2006, **26:**1579-1588.
9. Kaplun L, Tzirkin R, Bakhrat A, Shabek N, Ivantsiv Y, Raveh D: **The DNA damage-inducible UbL-UbA protein Ddi1 participates in Mec1-mediated degradation of Ho endonuclease.** *Mol Cell Biol* 2005, **25:**5355-5362.
10. Mizushima N, Noda T, Yoshimori T, Tanaka Y, Ishii T, George MD, Klionsky DJ, Ohsumi M, Ohsumi Y: **A protein conjugation system essential for autophagy.** *Nature* 1998, **395:**395-398.
11. Hirano Y, Yoshinaga S, Ogura K, Yokochi M, Noda Y, Sumimoto H, Inagaki F: **Solution structure of atypical protein kinase C PB1 domain and its mode of interaction with ZIP/p62 and MEK5.** *J Biol Chem* 2004, **279:**31883-31890.
12. Downes BP, Saracco SA, Lee SS, Crowell DN, Vierstra RD: **MUBs, a family of ubiquitin-fold proteins that are plasma membrane-anchored by prenylation.** *J Biol Chem* 2006, **281:**27145-27157.
13. Chano T, Ikegawa S, Saito-Ohara F, Inazawa J, Mabuchi A, Saeki Y, Okabe H: **Isolation, characterization and mapping of the mouse and human RB1CC1 genes.** *Gene* 2002, **291:**29-34.
14. Watanabe R, Chano T, Inoue H, Isono T, Koiwai O, Okabe H: **Rb1cc1 is critical for myoblast differentiation through Rb1 regulation.** *Virchows Arch* 2005, **447:**643-648.
15. Luders J, Demand J, Hohfeld J: **The ubiquitin-related BAG-1 provides a link between the molecular chaperones Hsc70/Hsp70 and the proteasome.** *J Biol Chem* 2000, **275:**4613-4617.

16. Sakata E, Yamaguchi Y, Kurimoto E, Kikuchi J, Yokoyama S, Yamada S, Kawahara H, Yokosawa H, Hattori N, Mizuno Y, et al: **Parkin binds the Rpn10 subunit of 26S proteasomes through its ubiquitin-like domain.** *EMBO Rep* 2003, **4:**301-306.
17. Nakamura M, Tanigawa Y: **Noncovalent interaction of MNSFbeta, a ubiquitin-like protein, with histone 2A.** *Comp Biochem Physiol B Biochem Mol Biol* 2005, **140:**207-210.
18. Tsukahara M, Suemori H, Noguchi S, Ji ZS, Tsunoo H: **Novel nucleolar protein, midnolin, is expressed in the mesencephalon during mouse development.** *Gene* 2000, **254:**45-55.
19. Liu S, Yu Y, An H, Li N, Lin N, Wang W, Zhang W, Wan T, Cao X: **Cloning and identification of a novel ubiquitin-like protein, BMSC-UbP, from human bone marrow stromal cells.** *Immunol Lett* 2003, **86:**169-175.
20. Gao YG, Song AX, Shi YH, Chang YG, Liu SX, Yu YZ, Cao XT, Lin DH, Hu HY: **Solution structure of the ubiquitin-like domain of human DC-UbP from dendritic cells.** *Protein Sci* 2005, **14:**2044-2050.
21. May MJ, Larsen SE, Shim JH, Madge LA, Ghosh S: **A novel ubiquitin-like domain in IkappaB kinase beta is required for functional activity of the kinase.** *J Biol Chem* 2004, **279:**45528-45539.
22. Zhao C, Denison C, Huibregtse JM, Gygi S, Krug RM: **Human ISG15 conjugation targets both IFN-induced and constitutively expressed proteins functioning in diverse cellular pathways.** *Proc Natl Acad Sci U S A* 2005, **102:**10200-10205.
23. Andersen JB, Strandbygard DJ, Hartmann R, Justesen J: **Interaction between the 2'-5' oligoadenylate synthetase-like protein p59 OASL and the transcriptional repressor methyl CpG-binding protein 1.** *Eur J Biochem* 2004, **271:**628-636.
24. Hipp MS, Kalveram B, Raasi S, Groettrup M, Schmidtke G: **FAT10, a ubiquitin-independent signal for proteasomal degradation.** *Mol Cell Biol* 2005, **25:**3483-3491.
25. Bayle J, Lopez S, Iwai K, Dubreuil P, De Sepulveda P: **The E3 ubiquitin ligase HOIL-1 induces the polyubiquitination and degradation of SOCS6 associated proteins.** *FEBS Lett* 2006, **580:**2609-2614.
26. Bradsher JN, Jackson KW, Conaway RC, Conaway JW: **RNA polymerase II transcription factor SIII. I. Identification, purification, and properties.** *J Biol Chem* 1993, **268:**25587-25593.
27. Garrett KP, Aso T, Bradsher JN, Foundling SI, Lane WS, Conaway RC, Conaway JW: **Positive regulation of general transcription factor SIII by a tailed ubiquitin homolog.** *Proc Natl Acad Sci U S A* 1995, **92:**7172-7176.
28. Kamura T, Sato S, Haque D, Liu L, Kaelin WG, Jr., Conaway RC, Conaway JW: **The Elongin BC complex interacts with the conserved SOCS-box motif present in members of the SOCS, ras, WD-40 repeat, and ankyrin repeat families.** *Genes Dev* 1998, **12:**3872-3881.
29. Kibel A, Iliopoulos O, DeCaprio JA, Kaelin WG, Jr.: **Binding of the von Hippel-Lindau tumor suppressor protein to Elongin B and C.** *Science* 1995, **269:**1444-1446.
30. Iliopoulos O, Levy AP, Jiang C, Kaelin WG, Jr., Goldberg MA: **Negative regulation of hypoxia-inducible genes by the von Hippel-Lindau protein.** *Proc Natl Acad Sci U S A* 1996, **93:**10595-10599.

31. Schmidtke G, Kalveram B, Weber E, Bochtler P, Lukasiak S, Hipp MS, Groettrup M: **The UBA domains of NUB1L are required for binding but not for accelerated degradation of the ubiquitin-like modifier FAT10.** *J Biol Chem* 2006, **281:**20045-20054.
32. Ko HS, Uehara T, Tsuruma K, Nomura Y: **Ubiquilin interacts with ubiquitylated proteins and proteasome through its ubiquitin-associated and ubiquitin-like domains.** *FEBS Lett* 2004, **566:**110-114.
33. McNally T, Huang Q, Janis RS, Liu Z, Olejniczak ET, Reilly RM: **Structural analysis of UBL5, a novel ubiquitin-like modifier.** *Protein Sci* 2003, **12:**1562-1566.
34. Wilkinson CR, Dittmar GA, Ohi MD, Uetz P, Jones N, Finley D: **Ubiquitin-like protein Hub1 is required for pre-mRNA splicing and localization of an essential splicing factor in fission yeast.** *Curr Biol* 2004, **14:**2283-2288.
35. Dittmar GA, Wilkinson CR, Jedrzejewski PT, Finley D: **Role of a ubiquitin-like modification in polarized morphogenesis.** *Science* 2002, **295:**2442-2446.
36. Holowaty MN, Sheng Y, Nguyen T, Arrowsmith C, Frappier L: **Protein interaction domains of the ubiquitin-specific protease, USP7/HAUSP.** *J Biol Chem* 2003, **278:**47753-47761.
37. Wyndham AM, Baker RT, Chelvanayagam G: **The Ubp6 family of deubiquitinating enzymes contains a ubiquitin-like domain: SUb.** *Protein Sci* 1999, **8:**1268-1275.
38. Kaye FJ, Modi S, Ivanovska I, Koonin EV, Thress K, Kubo A, Kornbluth S, Rose MD: **A family of ubiquitin-like proteins binds the ATPase domain of Hsp70-like Stch.** *FEBS Lett* 2000, **467:**348-355.
39. Kim JE, Kim SJ, Lee BH, Park RW, Kim KS, Kim IS: **Identification of motifs for cell adhesion within the repeated domains of transforming growth factor-beta-induced gene, betaig-h3.** *J Biol Chem* 2000, **275:**30907-30915.
40. Komatsu M, Chiba T, Tatsumi K, Iemura S, Tanida I, Okazaki N, Ueno T, Kominami E, Natsume T, Tanaka K: **A novel protein-conjugating system for Ufm1, a ubiquitin-fold modifier.** *Embo J* 2004, **23:**1977-1986.
